# Bronchitis, COPD, and pneumonia after viral endemic of patients with leprosy on Sorok Island in South Korea

**DOI:** 10.1007/s00210-023-02407-7

**Published:** 2023-02-11

**Authors:** Jong Hoon Lee, Badar Kanwar, Asif Khattak, Eric Altschuler, Consolato Sergi, So Jeong Lee, Su-Hee Choi, Jungwuk Park, Michael Coleman, Jean Bourbeau

**Affiliations:** 1 Science and Research Center, Seoul National University College of Medicine, 103 Daehak-ro, Jongno-gu, 03080 Seoul, Republic of Korea; 2 Department of Respiratory Medicine, Seoul Metropolitan Seobuk Hospital, 49 Galhyeon-ro 7-gil, Yeokchon-dong Eunpyeong-gu, Seoul, 03433 South Korea; 3 Department of Intensive Care Unit and Neonatal Intensive Care, Hunt Regional Hospital Greenville, Greenville, TX 75401 USA; 4 Physical Medicine/Rehab, Metropolitan Hospital, New York, NY 10029 USA; 5 Division of Anatomical Pathology, Children’s Hospital of Eastern Ontario (CHEO), University of Ottawa, 401 Smyth Road, Ottawa, ON K1H 8L1 Canada; 6 Department of BioSciences, Wiess School of Natural Sciences, Rice University, Houston, TX USA; 7 Department of Obstetrics and Gynaecology, Seoul National University Hospital, Seoul, Republic of Korea; 8 Research Center of Integrative Functional Medicine, Department of Neurosurgery, Chungdam Hospital, Seoul, Republic of Korea; 9 College of Health and Life Sciences, Aston University, Birmingham, B4 7ET UK; 10 Respiratory Epidemiology and Clinical Research Unit, McGill University Health Centre, Montréal, QC Canada

**Keywords:** Bronchitis, COPD, Pneumonia, Viral respiratory diseases, Dapsone, Acetylcholinesterase inhibitors (AChEIs), NMDA antagonist memantine

## Abstract

Viral respiratory diseases (VRDs) cause lung inflammation and inflammatory cytokine production. We study whether dapsone is responsible for its observed preventive treatment effects of the sustained viral RNA interferon response. Around 2008 and 2012, Korea’s Dementia Management Act stipulated drastic changes in the administration of dementia medication by medical staff. Participants were randomized and we compared leprosy patients with VRDs after prescribing dapsone as a standard treatment from 2005 to 2019. Significance was evaluated based on the dapsone-prescribed (+) subgroup and the dapsone-unprescribed (−) subgroup of the VRD diagnosed (+) and VRD undiagnosed (−) subgroup. We analyzed VRD ( +)/(− with dapsone (+)/(−) group and used a T-test, and designed the equation of acetylation with dapsone and acetylcholine (AA) equation. The 6394 VRD participants who received the dapsone intervention compared to the 3255 VRD participants in the control group demonstrated at T2 VRD (+) dapsone (−) (mean (*M*) = 224.80, SD = 97.50): T3 VRD (−) dapsone (+) (*M* = 110.87, SD = 103.80), proving that VRD is low when dapsone is taken and high when it is not taken. The *t* value is 3.10, and the *p* value is 0.004395 (significant at *p* < 0.05). After an increase in VRDs peaked in 2009, bronchitis, COPD, and pneumonia surged in 2013. The AA equation was strongly negatively correlated with the prevalence of bronchitis and chronic obstructive pulmonary disease (COPD): with bronchitis, *r*(15) =  −0.823189, *p* = 0.005519, and with COPD, *r*(15) =  −0.8161, *p* = 0.000207 (significant at *p* < 0.05). Dapsone treated both bronchitis and COPD. This study provides theoretical clinical data to limit acetylcholine excess during the VRD pandemic for bronchitis, COPD, and pneumonia.

**Supplementary Information:**

The online version contains supplementary material available at 10.1007/s00210-023-02407-7.

## Introduction

Sorok Island was established in May 1916 to quarantine leprosy patients. The public health report filed on June 4, 1946, increased the capacity of Sorokdo Leper Colony to between 8000 and 9000, making it the largest leprosarium in the world (Kim 2012; Jane 2010). Sister M. Stoeger and Sister M. Pissarek cared for the patients from 1962 to 2005 (Anthony 2019).

The antibiotic dapsone (4,4′-diaminodiphenyl sulfone, DDS) is predominantly associated with treating leprosy and is both an antibiotic and ANTI-inflammatory agent (Wolf et al. 2002). Dapsone has been used for leprosy, malaria, toxoplasmosis, and *Pneumocystis* pneumonia in persons with human immunodeficiency virus infection. Moreover, dapsone is prescribed for dermatitis herpetiformis, linear IgA dermatosis, bullous pemphigoid, subcorneal pustular dermatosis, erythema elevatum diutinum, bullous systemic lupus erythematosus, and other chronic inflammatory diseases characterized by the infiltration of neutrophils or eosinophils (Wozel 2010).

Acetylation of dapsone showed genetic polymorphism and reproducible individual characteristics. Acetylation of dapsone and deacetylation of monoacetyl dapsone occurred concurrently, and plasma ratios of acetylated to parent drug were attained constantly but characteristic for the individual (Gelber et al. 1971). Acetylation of aspirin inhibits cyclic GMP–AMP synthase (cGAS)-mediated interferon production, and cGAS acetylation on Lys384, Lys394, or Lys414 contributes to keeping cGAS inactive (Dai et al. 2019). The gut microbiota drives systemic antiviral immunity of type I interferon (IFN-I) priming. DNA-containing membrane vesicles from the gut microbiota were found in circulation. They promoted the clearance of both herpes simplex virus type 1 of DNA virus and vesicular stomatitis virus of RNA virus in a GAS-STING-IFN-I axis (Erttmann et al. 2022).

We investigated Hansen’s disease (HD) patients with dapsone following the Dementia Management Act (DMA), enacted in 2011, in Sorokdo National Hospital. We analyzed the medical records of Sorokdo National Hospital from 2005 to 2020. We compared the incidence of viral respiratory diseases (VRDs) with dapsone prescriptions for persistent lung inflammation, inflammatory cytokine production, viral RNA, and sustained IFN response; indeed, these responses are recapitulated and contribute to the pathology of severe acute respiratory syndrome coronavirus 2 (SARS-CoV-2) infection.

## Methods

### Study design

Medical data on the correlation between DDS and respiratory diseases were then analyzed by the International Classification of Diseases (ICD) codes of VRDs. There was no significant change and no statistical correlation (Lee et al. 2020a). However, in the study of the dapsone effect in antihistamine refractory chronic idiopathic urticaria, VRDs occurred in only three patients in the placebo group (Morgan et al. 2014). A higher dose of dapsone was required when the patient developed a tracheal infection, but the patient had no similar VRDs (Zheng et al. 2021). We correlated dapsone to compete with the NLRP3 inflammasome (Lee et al. 2020a). As NLRP3 plays a critical role in viral immunopathology (Malinczak et al. 2021), we analyzed the relationship between bacterial respiratory diseases (RDs) and VRDs (Fig. S1).

HD patients took dapsone for their lives but did not take dementia symptom improvement drugs. We included a control cohort from February 1962 to November 21, 2005 (Lee 2022b). Around 2008 and 2012, Korea’s Dementia Management Act (DMA) stipulated drastic changes in the administration of dementia medication by medical staff (Lee et al. 2022). It facilitated the EDI code-based cohort studies, randomized the cohort at a complete-blinded state, and made the RCT study provide causality (Lee et al. 2022).

DMA separated the dapsone-prescribing (+) group from the dapsone non-prescribing (−) group. Psychiatrists prescribed AAD instead of dapsone to treat mild cognitive impairment (MCI) or Alzheimer’s disease (AD). We connected the EMR database of the Sorokdo National Hospital, archived from January 2005 to June 2019, and searched the ICD-10 codes of RDs with dapsone. This cohort study is the second to be validated by RCT methodology because the intervention was performed for dementia treatment.

### Population demography

HD patients would spend their whole lives on Sorok Island. According to the request for disclosure of health checkup information from 2005 to 2020 on October 27, 2020, there were a total of 2186 people (1152 males, 1034 females), and the average age was 83.7 years (median (*M*) 84, interquartile range (IQR) 76.8–91.2, standard deviation (SD) 10.8, 95% confidence interval (CI): 0.45, 83.6–84.5) (Lee et al. 2022).

### Eligibility criteria

According to the Infectious Disease Control and Prevention Act, all Hansen subjects on Sorok Island have been registered and treated at Sorokdo National Hospital. This cohort consisted of HD patients, dapsone, and respiratory diseases in all Hansen subjects, according to the data received from Sorokdo National Hospital by South Korea’s Official Information Disclosure Act. We searched all medical records of the Sorokdo National Hospital with ICD-10 codes in South Korea from 2005 when the government computerized the codes.

Study Setting for ICD Code of Korean Diseases and Medicines (ICD-10 Version: 2019).

For Respiratory Diseases (Table S2): J20.9, J15, J15.8, J15.9, J17.0, J18.8, J18.9, J20.9, J30.0, J30.4, J31.0, J31.1, J31.2, J32, J32.0, J32.4, J32.8, J32.9, J34.0, J34.2, J34.8, J35.0, J36, J37.0, J38.0, J38.3, J39.0, J40, J42, J44, J44.9, J45, J45.0, J45.1, J45.9, J46, J47, J69.0, J81, J85.1, J90, J93, J94.2, J95.3, J96.0, J98.1, J98.8, J98.9.

For VRD (Table S2-1): J00, J02, J02.9, J03, J03.9, J04.0, J06.0, J06.9, J09, J10.8, J12.9, J20.9.

### Complete blinded study and randomization

HD patients have taken dapsone for their life or four types of dementia symptom improvement drugs: acetylcholinesterase inhibitors (AChEIs) and the NMDA antagonist memantine since 2008. The Korean government has established compulsory long-term care insurance (Chon 2014). The government successively established Community Dementia Reassurance Centers at all public Health Centers according to the National Duty for Dementia (Youn and Jeong 2018). In addition, medical teams reinforced the dementia management programs that administer AAD to MCI or AD patients as a preventive treatment (Lee et al. 2009; Ahn et al. 2015). The current mainstays of dementia treatment include AChEIs and memantine (Lee 2022b; Lee et al 2022). Ahn et al. (2015) insisted that the 1-year persistence rate of AChEIs should be precisely monitored to optimize treatment persistence for AD patients because patients are more likely to stop therapy than those in other countries. As a result, prescriptions for effective medications have increased. It overlaps with the enactment of the 2011 DMA. DMA significantly influenced the diagnosis and treatment of dementia. Medical staff treated HD patients with VRD or dementia, while no one knew about dapsone’s relationship with viral inflammasomes. This is a complete blinded randomized study by DMA.

### Interventions

According to the DMA, the medical staff of Sorokdo National Hospital started a full investigation in 2011 for the treatment of dementia for all HD patients on Sorok Island. As a result, AAD was prescribed for Hansen subjects diagnosed with dementia, and doctors stopped prescribing dapsone for inactive HD patients. They have followed up on all HD patients since 2011. As a result, DMA administered dapsone to the trial group, and we classified dapsone (−) subjects as the control group.

### Outcomes

Significance was evaluated based on a *p*-value of 0.05 in the DDS (+) subgroup and the DDS (−) subgroup of the VRD-diagnosed (+) subgroup and the VRD-undiagnosed (−) subgroup.

### Statistical analysis

We used the software programs Object-Relational DBMS and Google spreadsheet with SPSS. The Mann–Whitney U test, one-way repeated-measures ANOVA calculator, and post hoc Tukey honestly significant difference (HSD) test were applied. A significant T test was performed among the T1: DDS(+)/VRD(+), T2: DDS(−)/VRD(+), T3: DDS(+)/VRD(−), and T4: DDS(−)/VRD(−) groups.

## Results

Nine thousand six hundred forty-nine participants were randomized from 2005 to 2020 on Sorok Island. We performed primary (Tables S4–S4-11, Figs. S2–S6) and secondary (Tables S5-2–S5-13, Figs. S7–S11) analyses based on the *p* value. Because all the results were significant, we used primary data to report the results (Fig. 1).

**Fig. 1:**
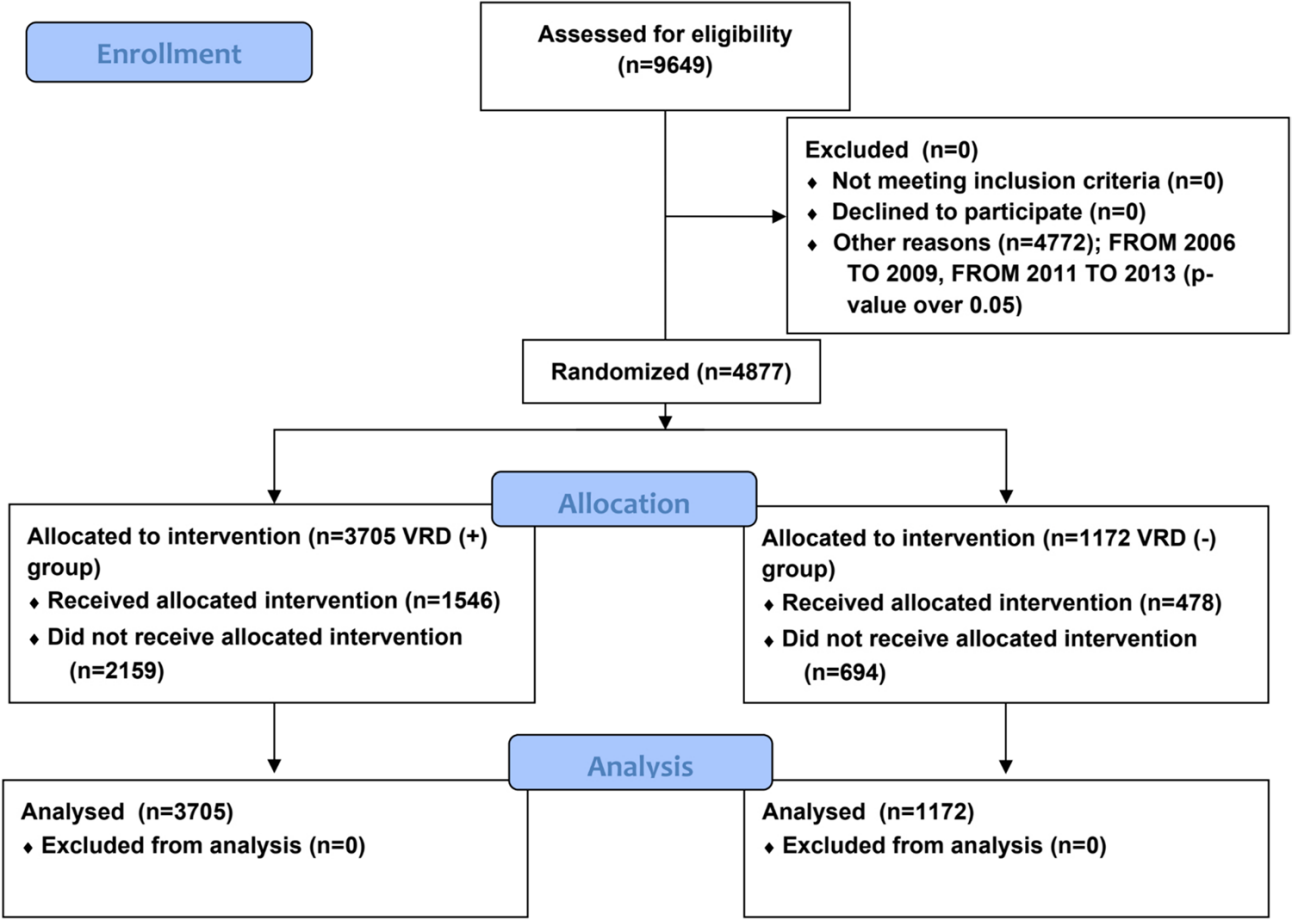
Flow chart of participants in viral respiratory disease infection on Sorok Island

VRD (+) subjects (Ss) (*S* = 6394, mean (*M*) = 426.27) consisted of the DDS (+) (*S* = 3022, *M* = 201.47) and DDS  −) groups (*S* = 3372, *M* = 224.80). VRD (−) subjects (*S* = 3255, *M* = 217.00) consisted of the DDS (+) (*S* = 1663, *M* = 110.87) and DDS (−) groups (*S* = 1592, *M* = 106.13).

The *f*-ratio value is 8.52. The *p* value in the one-way ANOVA calculator for independent measures is 0.000094 (Table S4-1). However, there were caveats to post hoc Tukey’s honestly significant difference. The pairwise comparisons (T1:T3, T1:T4, T2:T3, T2:T4, and T3:T4) were applicable except for T1:T2 and T2:T4 (Table S4-2) (Table 1).

**Table 1:** Viral respiratory disease (VRD) prevalence in the dapsone groups from 2005 to 2019

Year	T1^*^	T2^*^	T3^*^	T4^*^	Sum	Mean	SD^f^	95% CI^g^	[CI^g^	CI^g^]	*χ*2^*^	*p* value
2005	148	95	233	268	744	186	78.86	11.41	174.59	264.86	**13.5772**	**0.000229**
2006	166	111	237	208	722	180.5	54.74	8.05	172.45	235.24	*3.0793*	*0.079295*
2007	170	115	252	185	722	180.5	56.37	8.29	172.21	236.87	*0.2794*	*0.597118*
2008	207	128	219	148	702	175.5	44.34	6.61	168.89	219.84	*0.3293*	*0.566073*
2009	222	135	196	115	668	167	50.31	7.69	159.31	217.31	*0.0498*	*0.823418*
2010	202	170	186	105	663	165.75	42.55	6.54	159.21	208.30	**6.2203**	**0.012629**
2011	205	164	170	117	656	164	36.18	5.58	158.42	200.18	*0.8918*	*0.344987*
2012	237	211	103	102	653	163.25	70.95	10.97	152.28	234.20	*0.3981*	*0.528071*
2013	269	349	8	23	649	162.25	172.68	26.79	135.46	334.93	*3.7892*	*0.051583*
2014	236	349	6	32	623	155.75	164.85	26.16	129.59	320.60	**9.0547**	**0.00262**
2015	227	325	7	44	603	150.75	150.82	24.33	126.42	301.57	**14.7575**	**0.000122**
2016	207	319	4	61	591	147.75	142.63	23.25	124.50	290.38	**27.7773**	** < 0.00001**^**e**^
2017	193	301	14	55	563	140.75	131.44	21.96	118.79	272.19	**9.1835**	**0.002442**
2018	178	303	15	60	556	139	129.14	21.66	117.34	268.14	**8.2801**	**0.004008**
2019	155	297	13	69	534	133.5	123.66	21.21	112.29	257.16	**10.9433**	**0.000939**
Sum	3022	3372	1663	1592								
Mean	201.47	224.80	110.87	106.13								
SD	33.86	97.50	103.80	70.30								
95% CI	1.21	3.29	4.99	3.46								
[	200.26	221.51	105.87	102.68								
]	202.67	228.09	115.86	109.59								

The chi-square is 281.826

The *p* value is  <0.00001. I t is significant at *p* < 0.05

Bold means significant; italic, non-significant

^*^Four groups were classified: T1 group is DDS-prescribed (+) with VRD-diagnosed (+) subjects, T2 group is DDS-unprescribed (−) with VRD-diagnosed (+) subjects, T3 group is DDS-prescribed (+) with VRD-undiagnosed (−) subjects, and T4 group is DDS-unprescribed (−) with VRD-undiagnosed (−) subjects

^**^ Chi-square, e indicates a* p* value  <0.05, f standard deviation (SD), g confidence interval (CI)

*VRD*, viral respiratory disease

### *T* test

T1 (*M* = 201.47, SD = 33.86):T3 (*M* = 110.87, SD = 103.80) demonstrated that the VRD (+/−) groups in DDS (+) were clearly distinguished as of 2010. This describes that as of 2010, more people stopped taking dapsone. If HD subjects stopped taking dapsone, their condition would deteriorate because of exacerbated VRDs, and be hospitalized. We can find that the number of VRD patients was comparable (148 in 2005 and 155 in 2019). Very few people have been hospitalized for VRD in the group taking dapsone since 2013. The *t* value is 3.21, and the *p* value is 0.003287 (significant at *p* < 0.05) (Table S4-4, S4-5, and Fig. S3).

T1 (*M* = 201.47, SD = 33.86):T4 (*M* = 106.13, SD = 70.30) demonstrated that the number of people who took dapsone more increased since 2008 than those who did not take dapsone. This means that those who continued to take dapsone during the care of the two sisters from 1962 to 2005 began to understand the difference between taking dapsone and not taking it. After DMA in 2012 was enforced, there was the largest difference of 22 and 269 patients in 2013. The *t* value is 4.73, and the *p* value is 0.000058 (significant at *p* < 0.05) (Table S4-6, S4-7, and Fig. S4).

T2 (*M* = 224.80, SD = 97.50):T3 (*M* = 110.87, SD = 103.80) definitely proves that VRD is very low when dapsone is taken and very high when not taken. The *t* value is 3.10, and the *p* value is 0.004395 (significant at *p* < 0.05) (Table S4-8, S4-9, and Fig. S5).

The T2 (*M* = 224.80, SD = 97.50):T4 (*M* = 106.13, SD = 70.30) test confirms that the VRD increases when not taking dapsone. The* t* value is 3.82, and the *p* value is 0.000672 (significant at *p* < 0.05) (Table S4-10, S4-11, and Fig. S6).

**Fig. 2:**
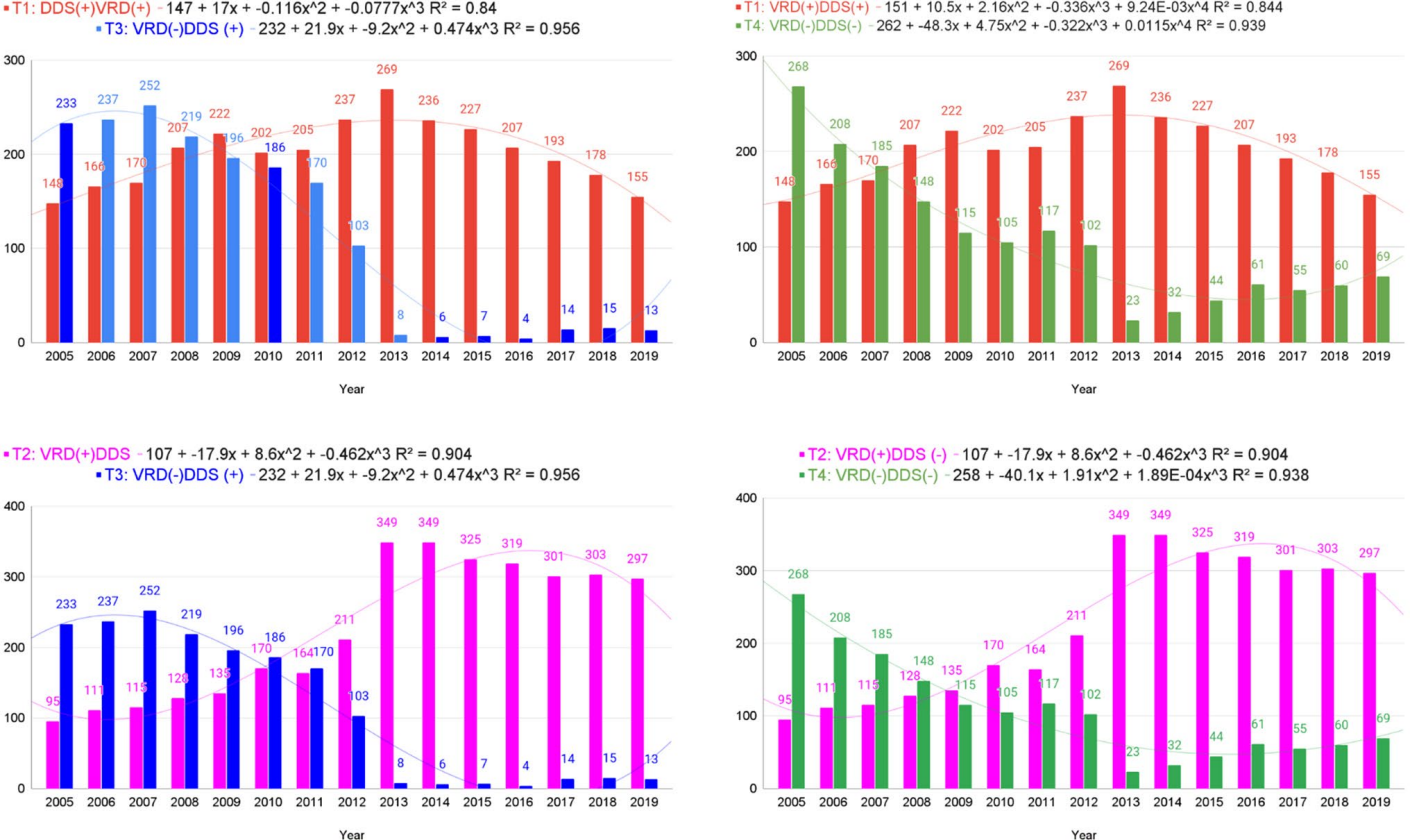
Study for T1, T2, T3, and T4. T1 group is dapsone prescribed with VRD-diagnosed subjects, T2 group is dapsone unprescribed with VRD-diagnosed subjects, T3 group is dapsone prescribed with VRD-undiagnosed subjects, and T4 group is dapsone unprescribed with VRD-undiagnosed subjects. After the Dementia Management Act was enacted in 2010, it became clear whether dapsone was prescribed because leprosy patients at Sorok Island should visit Sorokdo National Hospital to receive treatment. The column chart for the T test—changes started in 2010, and the VRD patterns are apparent in T1, T2, T3, and T4 from 2005 to 2019. The proportion of T2 patients without dapsone and with VRD increased significantly. T2:T3 graph shows that HD patients with prescribed dapsone have a very low prevalence of VRD. T1:T3 and T1:T4 demonstrate that VRD (+/−) groups in the dapsone (+) are distinguished as 2010. Furthermore, trend lines (*R*^2^ > 0.95) show significant relationships between the VRD (+/−) and DDS (+/−) groups. We plotted the trend line using a cubic polynomial equation, and the equation used was presented

T2:T3 and T2:T4 can explain no prevalence during the pandemic period of SARS-CoV (2002), influenza A virus subtypes H1N1 (2009), MERS (2015), and SARS-CoV-2 (2020) on Sorok Island. In addition, the T1:T3, T1:T4, T2:T3, and T2:T4 tests indicate that as of 2010, the group with dapsone (+) and the group without dapsone (−) were separated, and the group taking dapsone should have milder symptoms of VRD. SARS-CoV-2 as RNA-virus activates cGAS– stimulator of interferon genes (STING) signaling in endothelial cells through mitochondrial DNA release, which leads to type I IFN production, and pharmacological inhibition of STING reduces severe lung inflammation and disease severity (Domizio et al. 2022). This provides evidence that dapsone inhibits interferon, which is an exacerbation of the viral respiratory disease (Fig. 2).

### Immune and interferon-related respiratory diseases

We explored interferon-related diseases and classified those as (1) immune-related inflammatory diseases, (2) bronchitis, (3) bacteria-origin pneumonia, and (4) chronic obstructive pulmonary disease (COPD) from Tables S2, S2-1, and S2-2 (Table 2).

**Table 2:** Respiratory disease (RD) prevalence on Sorok Island

Disease name	ICD	2005	2006	2007	2008	2009	2010	2011	2012	2013	2014	2015	2016	2017	2018	2019
Asthma	J45	6	5	5	5	5	5	5	4	2	3	2	1	1	1	
Predominantly allergic asthma	J45.0			40	40											
Nonallergic asthma	J45.1			36												
Asthma, unspecified	J45.9									5	6	7	6	5	3	2
Status asthmaticus	J46				4	4	4									
Vasomotor rhinitis	J30.0			3	45	54	96	99	182	180	110	96	28	30	29	9
Allergic rhinitis, unspecified	J30.4	11	11	60	189	205	197	151	142	178	129	102	71	47	40	21
Chronic rhinitis	J31.0	10	40	40	16	95	91	64	19	15	18	19	16	14	11	1
Chronic sinusitis	J32				571	636	567	455	345	291	155	128		106		5
Chronic maxillary sinusitis	J32.0									1	9	20		17		5
Chronic pansinusitis	J32.4											2	2	4	4	4
Other chronic sinusitis	J32.8												1	1	1	1
Chronic sinusitis, unspecified	J32.9	11	19	18	16	15	15	44	41	42	33	32	32	29		
Chronic laryngitis	J37.0	6	6	6		7	6	6	6	5	7	12	15	11	8	3
Chronic nasopharyngitis	J31.1									2	17	33	49	38	30	8
Chronic pharyngitis	J31.2		27							79	168	226		188	130	
Abscess, furuncle, and carbuncle of nose	J34.0		1	1	122	127	59	59	43	24	51	47	47	41	25	16
Deviated nasal septum	J34.2				5	56	56	56	120	70	51	65	22	26	27	6
Other specified disorders of nose and nasal sinuses	J34.8	2	4		4	4	4	3	3	5	5	7	6	2	2	2
Chronic tonsillitis	J35.0										5	10	12	7	4	2
Peritonsillar abscess	J36								7	7	7	7	7	7		
Retropharyngeal and parapharyngeal abscess	J39.0								16	16	16					
Immune related inflammatory diseases		47	114	210	1017	1224	1116	956	951	945	804	829	329	584	325	85
Acute bronchitis, unspecified	J20.9	11	11	1	1	1	1	1	515	675	655	354	225	222	219	4
Acute bronchitis, unspecified	J20.9	11	11	1	1	1	1	1	515	675	655	354	225	222	219	4
Bronchitis, not specified as acute or chronic	J40	4	4	4	4	4	4	4	4	1	1					
Unspecified chronic bronchitis	J42	11	58	28	41	41	41	30	13	13						
Bronchitis		37	84	34	47	47	47	36	1047	1364	1311	708	450	444	438	8
Bacterial pneumonia, NEC	J15			1	18	24	18	143	17	6	2					
Other bacterial pneumonia	J15.8		16		86	89	88	3	3	3			1	16	7	1
Bacterial pneumonia, unspecified	J15.9	5	5	5	95	5	6	136	258	638	218	68	32	5	4	4
Pneumonia in bacterial diseases classified elsewhere	J17.0		20	20	20	20	20	20	20	20	20					
Other pneumonia, organism unspecified	J18.8		17		111	111	110	110	110	127	17	17	17			
Pneumonia, unspecified	J18.9	36	36	36	36	15	7	7	7	7	9	16	23	18	13	8
Pneumonia		41	94	62	366	264	249	419	415	801	266	101	73	39	24	13
PNEUMONITIS DUE TO FOOD AND VOMIT	J69.0		5	5	6	6	1	9	61	89	34	39	36	35	34	1
Other chronic obstructive pulmonary disease	J44				65	64	64	64								
Chronic obstructive pulmonary disease, unspecified	J44.9	35	29	144	242	356	404	382	1257	1447	948	631	473	375	212	
Bronchiectasis	J47	2	2	2	2	2	2	2	2	3	3	2	2	2		
COPD	37	31	146	309	422	470	448	1259	1450	951	633	475	377	212	0	
Pulmonary Edema	J81											3	2	2	2	1
Abscess of lung with pneumonia	J85.1		5	5	5											
Pleural effusion, NEC	J90											2	1	2		
Pneumothorax	J93	1	5	1	1	1	1	1	1	1	1	1				
Hemothorax	J94.2										2	1	1			
Chronic pulmonary insufficiency following surgery	J95.3											1		1		
Acute respiratory failure	J96.0				20											
Pulmonary collapse	J98.1											1				
Other specified respiratory disorders	J98.8													1		
Respiratory disorder, unspecified	J98.9													5	2	1
End stage		1	10	6	26	1	1	1	1	1	3	9	4	11	4	2

Immune-related inflammatory diseases showed a sharp increase in prevalence from 2008 to 2015, followed by a decrease. COPD increased slowly from 2008 to 2011, rapidly increased in 2012 and 2013, and decreased. Bronchitis levels rose rapidly from 2012 to 2014 and then decreased. Pneumonia increased sharply in 2013 compared to previous years.

Since 2008, immune-related inflammatory diseases have increased rapidly. It is a period of viral respiratory disease: 2008–2010 endemic on Sorok Island. Since 2012, COPD has increased, as have bronchitis and pneumonia frequencies. We observed a decrease in the average age of death in the group taking AAD and psychotropic drugs from 2008 to 2015 (Lee 2022b) (Fig. S12). We investigated asthma and lung function trajectories leading to COPD from the ICD-10 codes asthma (J45), predominantly allergic asthma (J45.0), nonallergic asthma (J45.1), asthma, unspecified (J45.9), and status asthmaticus (J46) and COPD patients: other chronic obstructive pulmonary disease (J44), chronic obstructive pulmonary disease, unspecified (J44.9), and bronchiectasis (J47) to identify the prevalence of asthma–COPD relationships. The prevalence of asthma–COPD was not associated at all. However, we only observed a much higher prevalence of COPD than asthma. Asthma and lung function trajectories did not lead to COPD (Fig. S13).

### Factors from the number of diagnosed Alzheimer’s disease patients and dapsone use group

Because pharmaceutical companies that produce AChEI reported its frequent side effects like pharyngitis, pneumonia, increased cough, and bronchitis (Lee et al. 2020b), and AAD use in dementia-related disorders increased mortality (Stone 2005; Jong Hoon 2022), we used the pile-up data from Sorok Island_Cohort-Lee, Jong Hoon (2022), “Basic cohort study: dapsone is an anticatalysis for AD exacerbation,” Mendeley Data, V2 (Data S1).

We formulated the factor to calculate the relationship between acetylation and acetylcholine.

[Acetylation-acetylcholine (AA) equation]:

1 $$\mathrm{The}\;\mathrm{Dapsone}\;\mathrm{IFN}1\;\mathrm{factor}=\left|\;\mathrm{DDS}\;\left(+\right)-\mathrm{sum}\;\mathrm{AD}\;(+)\;\right|$$

A total was calculated for all the people taking dapsone and all the individuals diagnosed with AD taking AAD, subtracting 1 from the other, processing the data as an absolute value (Table S6). We used the Pearson correlation coefficient calculator and Spearman’s rho calculator to correlate the factors and the prevalence of bronchitis, pneumonia, and COPD.

Our calculations can be summarized as follows:

Pearson correlation coefficient calculator

The AA equation and bronchitis were strongly negatively correlated, *r*(15) =  − 0.823189, *p* = 0.005519. The result is significant at *p* < 0.05.

The AA equation and pneumonia variables were weakly and negatively correlated, *r*(15) =  − 0.4402, *p* = 0.100742. Therefore, the result is not significant at *p* < 0.05.

The AA equation and COPD were found to be strongly negatively correlated, *r*(15) =  − 0.8161, *p* = 0.000207. The result is significant at *p* < 0.05.

**Fig. 3:**
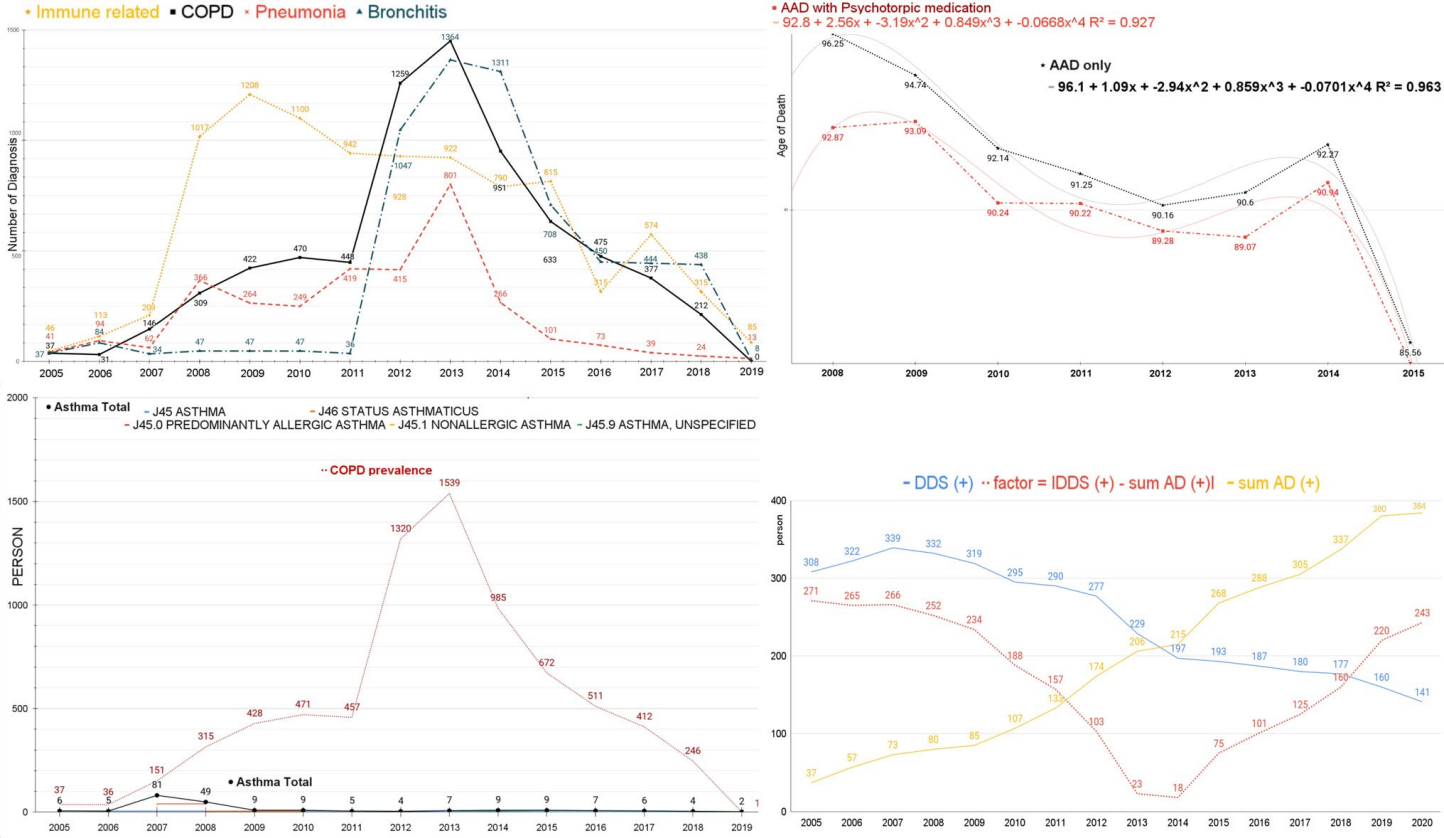
Bronchitis, COPD, and pneumonia after the endemic of viral respiratory diseases. (1) The initial step in the cellular entry of viral respiratory disease (VRD) is binding the spike protein to cell surface receptors. This allows the fusion of the virus to the cell surface through cellular proteases such as TMPRSS2 and furin to be involved in priming the spike protein. Virions are taken up into endosomes, where the virus may be cleaved and possibly activated by the cysteine protease. The virus uses endogenous cellular machinery to replicate itself inside the cell (Muniyappa and Gubbi 2020). Pathogen-associated molecular patterns (PAMPs) or danger-associated molecular patterns (DAMPs) induced by the virus may affect respiratory symptoms. The prevalence of viral respiratory diseases showed a sharp increase from 2008, followed by COPD and bronchitis, increasing in 2012 and decreasing from 2014. Pneumonia increased sharply in 2013 compared to previous years. (2) The expectancy of Hansen’s disease (HD) patients with Alzheimer’s disease (AD) taking AChEIs or memantine with psychotropic medicines at Sorok Island. The mean ages of death decrease. The mean ages of deaths without taking additional psychotropic drugs are black. The life expectancy trends of HD patients taking other psychotropic medications (red) decreased. The life expectancy trends of HD patients taking anti-Alzheimer’s disease drugs (AAD) were decreased on Sorok Island (black), and those taking AAD with psychotropic medicines (red) were reduced more (Lee 2022b). (3) We reviewed the prevalence of asthma patients from the ICD-10 codes ASTHMA (J45), PREDOMINANTLY ALLERGIC ASTHMA (J45.0), NONALLERGIC ASTHMA (J45.1), ASTHMA, UNSPECIFIED (J45.9), and STATUS ASTHMATICUS (J46). (4) We calculated that all Hansen’s disease patients were taking dapsone, and all the individuals diagnosed with Alzheimer’s disease (AD)^*^ were taking anti-Alzheimer’s disease (AAD)^**^. Then, we subtracted one from the other, processed the data as an absolute value, and illustrated these data graphically. Our results were strongly negatively correlated with COPD and bronchitis, not pneumonia

Spearman’s rho calculator gave the same result: the association between the two variables of bronchitis and COPD with the AA equation was considered statistically significant by normal standards. Pneumonia would not be considered statistically (Supplement S6.1, S6.2, and S6.3) (Fig. 3).

The AA equation was correlated with the prevalence of bronchitis and COPD. This means that dapsone treated and AAD exacerbated them, but dapsone not with pneumonia caused by bacteria. Ameliorating viral disease with dapsone (Lee et al. 2020a; Kanwar et al. 2021, 2022) or the downstream IFN-stimulated cascade with anti-IFNAR2 in the onset stages of disease (Lee 2022a) must attenuate overactive immune-mediated respiratory inflammatory diseases.

## Limitations

The limitation is that this study was conducted in an island area and on HD patients. Since dapsone’s maximal allowance price in South Korea was very low in 2016, pharmaceutics, which produced it in Korea, stopped the production of dapsone except for the supply for HD patients (Lee 2021). More studies are required to compare COVID-19 survival rates later.

## Discussion

### We recommend taking dapsone continuously for Hansen’s disease patients if there are no side effects

Dapsone activates specific T cells of hypersensitive patients expressing the risk allele HLA‐B* 13: 01. HLA-B*13:01-CD8^+^ T cells (cytotoxic T lymphocytes) induce a dapsone-responsive immune response (Zhao et al. 2019). The multidrug therapy containing rifampin and clofazimine with dapsone was decisive for treating leprosy (Ramos-e-Silva and Rebello 2001). According to our survey, some HD patients on Sorok Island have taken dapsone for over 20 years. Remarkably, we noted that some people took dapsone for more than 50 years (Lee 2022b; Lee et al. 2022; Kanwar et al. 2021). Therefore, if there are no dapsone side effects, we recommend taking dapsone continuously.

### COPD was associated with AChEIs and IFN1

COPD was associated with incident nonamnestic MCI in a dose-dependent manner in the Mayo Clinic Study on Aging (Singh et al. 2014). The risk of COPD exacerbation may increase in the first 90 days of AChEI therapy in patients with dementia and COPD (Mahan and Blaszczyk 2016). Virus infection and interferon treatment decreased the M2 muscarinic receptor gene expression on the parasympathetic nerve endings by causing the release of IFN-gamma, which inhibits M2 receptor gene expression (Jacoby et al. 1998). Acetylcholine excess appears to inhibit acetylcholine receptors for interferon production against virus invasion. The genesis of acetylcholine receptor needs interferon (Balasa et al. 1997). The muscarinic and nicotinic acetylcholine receptors play critical roles in regulating immune function (Kawashima et al. 2012). This study elucidates the correlation between donepezil and acetylcholine and suggests that acetylcholine excess negatively affects acetylcholine receptor gene expression.

An asthma component might facilitate the identification of COPD patients with no previous diagnosis of obstructive lung disease. The prevalence of asthma–COPD overlap syndrome was only 6% of the COPD patients who fulfilled both criteria (Baarnes et al. 2017). Our study also corresponds to previous results. Asthma and lung function trajectories did not lead to COPD.

The molecule neuropilin-1 (NRP1) plays an important and complex role in the secondary CD8 T-cell response to control viral infections and tumors (Hwang et al. 2019). We can divide innate lymphoid cells (ILCs) into three groups based on distinct cytokine secretion profiles and dependent transcription factors. Group 3 ILCs (ILC3s) are present in smokers and patients with COPD. ILC3s with NRP1 produce higher levels of cytokines than ILC3s without NRP1 (Shikhagaie et al. 2017). NRP1^+^ ILC3s play a potential role in inflammation and vascularization (Meininger et al. 2020). Dapsone might control the NRP1-ILC inflammatory pathway with the IFN1 or cGAS-STING cascade pathways through acetylation-deacetylation.

## Conclusion

This study provides theoretical clinical data to limit acetylcholine excess during the VRD pandemic for bronchitis, COPD, and pneumonia.

## Supplementary Information

Below is the link to the electronic supplementary material.

Supplementary file1 (DOCX 2542 KB)

## Data Availability

All data are available in the main text or the supplementary materials. Additional data supporting this study’s findings are available from the corresponding author upon reasonable request. In addition, the complete detailed survey is provided as a separate file. Lee, Jong Hoon (2022), “Data of viral respiratory diseases on Sorok Island during the pandemic”, Mendeley Data, V1, https://doi.org/10.17632/cwxswnjnb2.1.
