# Supplementary material for: Bronchitis, COPD, and pneumonia after viral endemic of patients with leprosy on Sorok Island in South Korea
Source: Naunyn Schmiedebergs Arch Pharmacol. 2023 Feb 11;396(7):1501–11. doi: 10.1007/s00210-023-02407-7 (PMC9918834; doi:10.1007/s00210-023-02407-7)
Supplement: Supplementary file 1 — Supplementary file1 (DOCX 2542 KB) [file 210_2023_2407_MOESM1_ESM.docx]

Online Supplementary Materials

for

Bronchitis, COPD, and Pneumonia after viral Endemic of patients with leprosy on Sorok Island in South Korea

Materials and Methods

Supplementary Text

Figs. S1 to S13

Tables S1 to S6

[**Statistical Analysis Plan** 4](#_Toc114943957)

[**Section 1. Administrative Information** 4](#_Toc114943958)

[**Section 2. Introduction** 5](#_Toc114943959)

[2.1 Background and Rationale 5](#_Toc114943960)

[2.2. Objectives 5](#_Toc114943961)

[2.3. Statistical Principles 5](#_Toc114943962)

[2.3.1. Confidence intervals and P values 5](#_Toc114943963)

[2.3.2. Protocol deviations 6](#_Toc114943964)

[2.4. Institutional permissions 6](#_Toc114943965)

[**Section 3. Study of DDS Group** 7](#_Toc114943966)

[3.1. Study Method of Sorok Island 7](#_Toc114943967)

[3.1.1. Cohort Design of Sorok Island 7](#_Toc114943968)

[Fig. S1. Cohort Design of Sorok Island - This retrospective cohort study was conducted on Sorok Island from 2005 to 2020. 8](#_Toc114943969)

[3.1.2. Complete blinded randomization 9](#_Toc114943970)

[3.2. Analysis of Viral Respiratory Diseases Cohort 9](#_Toc114943971)

[3.2.1. Study Setting for ICD-10 code 9](#_Toc114943972)

[Table S2. ICD-10 Study Code for Respiratory Diseases 9](#_Toc114943973)

[Table S2-1. ICD-10 Study Code for Viral Respiratory Diseases 12](#_Toc114943974)

[Table S2-2. ICD-10 Study Code for Viral Respiratory Diseases 13](#_Toc114943975)

[3.2.2. Statistical framework 15](#_Toc114943976)

[Section 4. Primary T test 16](#_Toc114943977)

[4.1. Primary T test for T1:T2:T3:T4 16](#_Toc114943978)

[Table S4. Primary T1:T2:T3:T4 study for the retrospective cohort study at Sorok Island. 16](#_Toc114943979)

[Table S4-1. One-way ANOVA Calculator for Independent Measures of Primary T1:T2:T3:T4 study for the retrospective cohort study at Sorok Island 16](#_Toc114943980)

[Table S4-2. Post hoc Tukey HSD 17](#_Toc114943981)

[Table S4-3. One-way ANOVA Calculator for repeated Measures of Primary T1:T2:T3:T4 study for the retrospective cohort study at Sorok Island 17](#_Toc114943982)

[Fig. S2. Study Results of Primary T1:T2:T3:T4 18](#_Toc114943983)

[4.2. T test for Primary T1:T3 18](#_Toc114943984)

[Table S4-4. Primary T test for T1:T3 18](#_Toc114943985)

[Table S4-5. Effect Size Calculator for T Test for T1:T3 19](#_Toc114943986)

[Fig. S3. Primary Study Result of T1:T3 19](#_Toc114943987)

[4.3. T test for Primary T1:T4 21](#_Toc114943988)

[Table S4-6. Primary T test for T1:T4 21](#_Toc114943989)

[Table S4-7. Effect Size Calculator for T Test for T1:T4 21](#_Toc114943990)

[Fig. S4. Primary Study Results of T1:T4 21](#_Toc114943991)

[4.4. T test for Primary T2:T3 23](#_Toc114943992)

[Table S4-8. Primary T test for T2:T3 23](#_Toc114943993)

[Table S4-9. Effect Size Calculator for T Test for T2:T3 23](#_Toc114943994)

[Fig. S5. Primary Study Results of T2:T3 24](#_Toc114943995)

[4.5. T test for Primary T2:T4 25](#_Toc114943996)

[Table S4-10. Primary T test for T2:T4 25](#_Toc114943997)

[Table S4-11. Effect Size Calculator for T Test for T2:T4 25](#_Toc114943998)

[Fig. S6. Primary Study Results of T2:T4 25](#_Toc114943999)

[**Section 5. Statistical Renormalization Analysis for the cohort study on Sorok Island** 27](#_Toc114944000)

[Table S5-1. Renormalization Study for Respiratory Diseases in Sorok Island 27](#_Toc114944001)

[5.1. T1:T2:T3:T4 Renormalization study for comparative analysis 29](#_Toc114944002)

[Table S5-2. The renormalization of the T1:T2:T3:T4 study at Sorok Island. 29](#_Toc114944003)

[Table S5-3. T1:T2:T3:T4 study for 2005, 2010, 2014-2019 Dealing Dependent variables at Sorok Island 29](#_Toc114944004)

[Table S5-4. One-Way ANOVA Calculator for Independent Measures of T1:T2:T3:T4 study for 2005, 2010, 2014-2019 at Sorok Island 30](#_Toc114944005)

[Table S5-5. Post hoc Tukey HSD 30](#_Toc114944006)

[Table S5-6. One-Way ANOVA Calculator for repeated Measures of T1:T2:T3:T4 study for 2005, 2010, 2014-2019 Dealing Dependent variables at Sorok Island 31](#_Toc114944007)

[Fig. S7. Dependent Study Result of T1 T2 T3 T4 31](#_Toc114944008)

[5.2. Addressing dependent variables for the T1:T3 study at Sorok Island 32](#_Toc114944009)

[Table S5-7. T test for T1:T3 32](#_Toc114944010)

[Table S5-8. Effect Size Calculator for T Test for T1:T3 32](#_Toc114944011)

[Fig. S8. Dependent Study Result of T1 T3 33](#_Toc114944012)

[Table S5-8. T test for T1:T4 33](#_Toc114944013)

[Table S5-9. Effect Size Calculator for T Test for T1:T4 34](#_Toc114944014)

[Fig. S9. Dependent Study Result of T1:T4 35](#_Toc114944015)

[Table S5-10. T test for T2:T3 35](#_Toc114944016)

[Table S5-11. Effect Size Calculator for T Test for T2:T3 36](#_Toc114944017)

[Fig. S10. Dependent Study Result of T2 T3 36](#_Toc114944018)

[Table S5-12. T test for T2:T4 37](#_Toc114944019)

[Table S5-13. Effect Size Calculator for T Test for T2:T4 37](#_Toc114944020)

[Fig. S11. Dependent Study Result of T2 T4 37](#_Toc114944021)

[5.3. The average age of death on Sorok Island. 38](#_Toc114944022)

[Fig. S12. The average age of death on Sorok Island. 38](#_Toc114944023)

[**Section 6. Statistical Analysis for the Correlation OR Coefficient of determination** 40](#_Toc114944024)

[6.1 Bronchitis and Factor 40](#_Toc114944025)

[6.2. Pneumonia and Factors 43](#_Toc114944026)

[6.3. COPD and Factor 45](#_Toc114944027)

[Table S6. The Factor for **Correlation Coefficient Determination.** 48](#_Toc114944028)

[Fig. S13. Asthma and lung function trajectories leading to COPD 50](#_Toc114944029)

[**Section 7. Safety** 51](#_Toc114944030)

**Statistical Analysis Plan**

1. Administrative Information

Principal Investigators: Jong-hoon Lee

Statistics Calculators of SPSS: https://www.socscistatistics.com/tests/

Contributor: Statistical responsible: Jong Hoon Lee^1^

^1^Science & Research Center, Seoul National University College of Medicine, Seoul, South Korea

**Section 1. Administrative Information**

The Korea National Institute for Bioethics Policy (KoNIBP) approved this study to properly manage life-sustaining treatment (approval number P01-202007-22-006).

Funder: None

Principal Investigators: Jong-hoon Lee

Statistics Calculators of SPSS: https://www.socscistatistics.com/tests/

The content of this Statistical Analysis Plan meets the requirements stated by the US Food and Drug Administration and conforms to the American Statistical Association's Ethical Guidelines. This protocol was organized following the guidelines proposed in the following:

Gamble C, Krishan A, Stocken D, Lewis S, Juszczak E, Doré C, et al. Guidelines for the Content of Statistical Analysis Plans in Clinical Trials. JAMA. 2017;318: 2337–2343. PMID: 29260229

**Section 2. Introduction**

2.1 Background and Rationale

The identification of safe, effective treatment for individuals with mild or moderate COVID-19 acute respiratory distress syndrome (ARDS) that prevents disease progression would reduce mortality. Dapsone is being evaluated for SARS-CoV-2 ARDS prevention and COVID-19-related diseases. Recent evidence has indicated that broad-spectrum dapsone may have some activity against the SARS-CoV-2 inflammasome of coronaviruses. A randomized controlled trial (RCT) in the intensive care unit (ICU) will demonstrate reduced viral inflammasome load among patients, such as familial Mediterranean fever (FMF), caused by homozygous or compounded heterozygous gain-of-function mutations in the Mediterranean fever gene, which encodes pyrin, an inflammasome protein. It is a hereditary autoinflammatory disease that presents with recurrent febrile attacks and polyserositis. Pyrin—the protein involved in FMF—has a role in activating the proinflammatory cytokine interleukin (IL)-1β, and colchicine is the only known treatment in this disease, but nearly 5–10% of patients are resistant to colchicine. In five of ten patients prescribed DDS, FMF attacks did not occur during at least six months (mean, eight months and six days), with a colchicine efficacy of 80% and a 50% response. Thus, DDS may be helpful as an alternative therapy in some, especially colchicine-resistant patients.

The Sorokdo National Hospital is responsible for the treatment of respiratory infectious diseases and has reported no prevalence of each pandemic of SARS-CoV (2002), influenza A virus subtype H1N1 (2009), MERS (2015), or SARS-CoV-2 (2020). Furthermore, the Sorokdo National Hospital reported a relationship between Dapsone and AD in Sorok Island from January 2020 to June 2021. Under the Information Disclosure Act, the National Sorokdo Hospital responded as follows in the response form to the request for information disclosure on April 15, 2021—7767726:

Table S1.

| 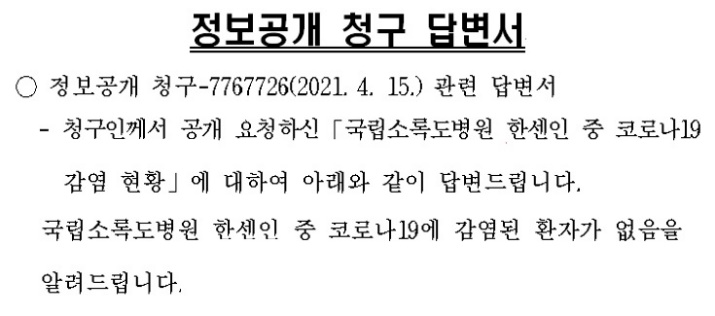 | "Regarding the "COVID-19 infection status among Hansen people of Sorokdo National Hospital" requested by the claimant (Number-7767726-2021/4/15), we will respond as follows: We are pleased to inform you that there are no patients infected with COVID-19 among Hansen Disease patients at National Sorokdo Hospital". |
| --- | --- |
| Original Information Disclosure Request - 7767726 (2021.4.15) Response | Translated Information Disclosure Request - 7767726 (2021.4.15) Response |

2.2. Objectives

To study the targeting of Nod-like receptor family pyrin domain-containing 3 (NLRP3) itself or up-/downstream factors of the NLRP3 inflammasome by Dapsone should be responsible for its observed preventive treatment effects, functioning as a competitor.

2.3. Statistical Principles

2.3.1. Confidence intervals and P values

The trial will report 95% confidence intervals and permutation P values estimated from the primary analysis.

2.3.2. Protocol deviations

The analysis population will be an intention to treat and will include all participants enrolled and randomized. Adherence to the protocol will be determined during the cross-sectional assessment based on patient reports of whether they took the medication by the trial. At that time, the trial will assess whether patients have taken other medications during the study period, including dapsone or other medicines not provided by the trial. Patients who take Dapsone not provided by the study will be considered protocol deviations but will be included according to the randomized group in the intention-to-treat analysis.

- 1. Institutional permissions

The Korea National Institute for Bioethics Policy (KoNIBP) approved this study to properly manage life-sustaining treatment (approval number P01-202007-22-006).

The KoNIBP approved the observational study of patients ethically based on FDA guidelines following the World Medical Association Declaration of Helsinki. Therefore, we carried out all methods following relevant ethical guidelines and regulations and reported the study results. In addition, Sorokdo National Hospital obtained informed consent from all participants under 18 from a parent or legal guardian.

**Section 3. Study of DDS Group**

- 1. Study Method of Sorok Island
     1. Cohort Design of Sorok Island

Sorok Island was established in May 1916 to quarantine leprosy patients as a Japanese leper colony. During the colonial period, the notorious expansion project for the Sorok Leprosarium was supposed from 1933 to 1941 because it competed with the Culion Leprosarium in the American-Occupied Philippines. The public health report filed on June 4, 1946, succinctly stated that they would increase the capacity of Sorokdo Leper Colony to 8,000 – 9,000 and make it the largest leprosarium in the world (*1, 2*).

However, it was not easy for those patients to see a doctor. Therefore, they self-administer a prescribed medication steadily. Finally, the missionaries went to Sorok Island to care for the leprosy patients.

This study is a cohort study with a history of more than 100 years. This study analyzed the medical records of Sorokdo National Hospital from 2005 to 2020. Sister M. Stoeger and Sister M. Pissarek cared for the patients for forty years(*3*). Therefore, we regarded those as a kind of control group initiated from 1962 to 2005. After graduating from the University of Innsbruck Nursing School in Tyrol, Western Austria, Sister Marianne Stoeger, who worked at a hospital in Innsbruck, joined Sorok Island in February 1962. Sister Margaritha Pissarek entered Sorok Island in October 1967. They left Sorok Island on November 21, 2005.

After President Dae-Jung Kim came to power in 1998, Hansen’s disease (HD) patients were given freedom. Many were devout Christians, but respiratory infectious diseases increased in a typical rural village as more and more became smokers. Sorok Island has become a tourist destination, and patients have come and gone freely.

Medical data on the correlation between DDS and respiratory diseases were then analyzed, but SARS-CoV (2002), influenza A virus subtype H1N1 (2009), MERS (2015), and SARS-CoV-2 (2020) did not occur. International Classification of Diseases (ICD) codes of respiratory infectious diseases (RIS) are J00 (acute nasopharyngitis), J01 (acute sinusitis), J01.8 (other acute sinusitis), J02 (acute pharyngitis), J02.9 (acute pharyngitis, unspecified), J03 (acute tonsillitis), J03.0 (streptococcal tonsillitis), J03.9 (acute tonsillitis, unspecified), J04.0 (acute laryngitis), J06.0 (acute laryngopharyngitis), J06.9 (acute upper respiratory infection, unspecified), J09 (Influenza due to identified zoonotic or pandemic influenza virus), J10.8 (Influenza with other manifestations, seasonal influenza virus identified), J12.9 (viral pneumonia, unspecified), J15.8 (other bacterial pneumonia), J15.9 (bacterial pneumonia, unspecified), J18.0 (bronchopneumonia, unspecified), J18.9 (pneumonia, unspecified), J20.9 (acute bronchitis, unspecified), J31 (chronic rhinitis, nasopharyngitis and pharyngitis), J31.0 (chronic rhinitis), J31.1 (chronic nasopharyngitis), J31.2 (chronic pharyngitis), J32 (chronic sinusitis), J32.4 (chronic pansinusitis), J32.8 (other chronic sinusitis), J35. 0 (chronic tonsillitis), and J37.0 (chronic laryngitis) (*4*).

Therefore, we connected to the EMR database of the Sorokdo National Hospital, archived from January 2005 to June 2019, and searched the ICD-9 and -10 codes of viral respiratory diseases (VRDs) with DDS.


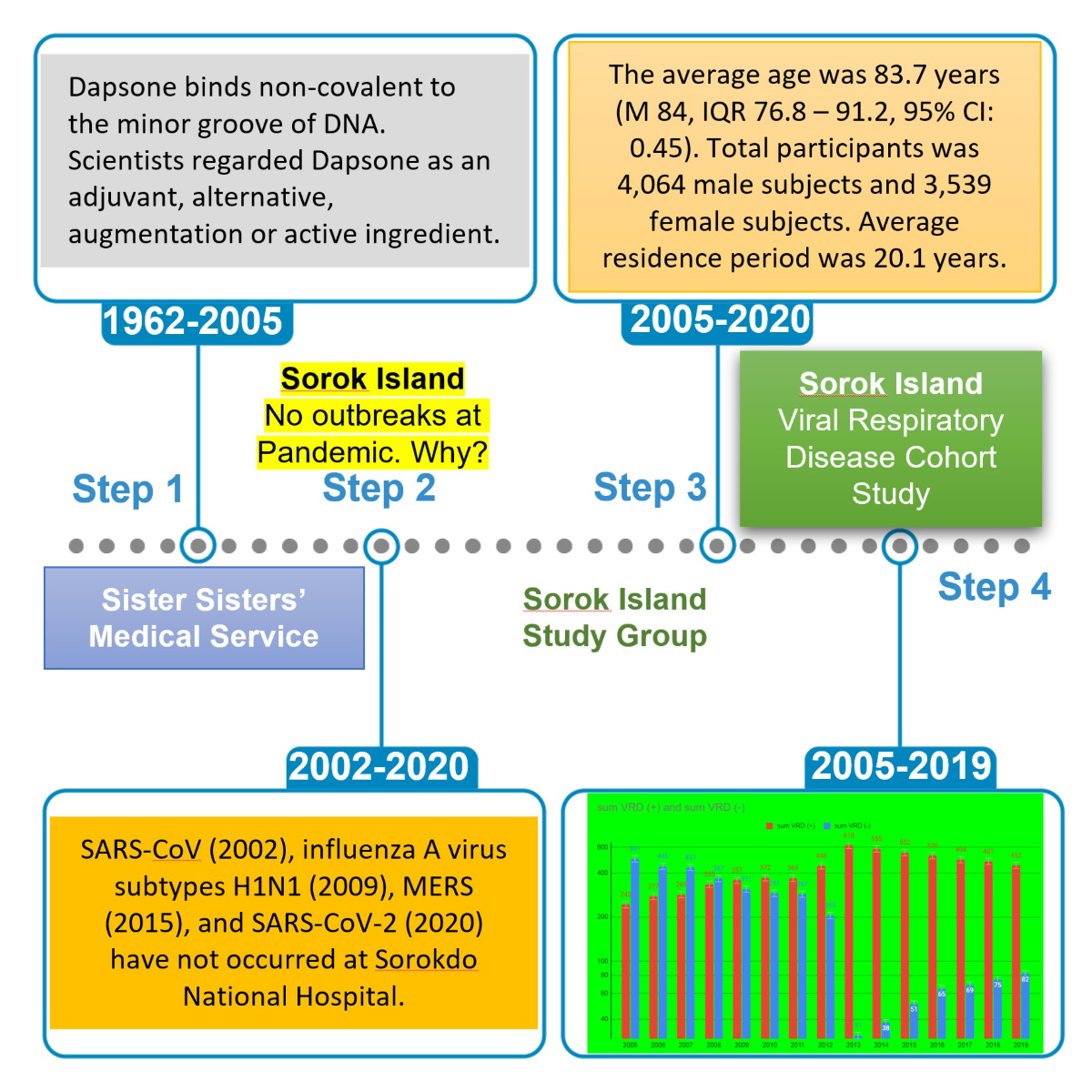


### Fig. S1. Cohort Design of Sorok Island - This retrospective cohort study was conducted on Sorok Island from 2005 to 2020.

There were 85 ICD respiratory disease codes in Sorokdo National Hospital. Therefore, a total of 54323 ICD codes from 2005 to 2019, when computerization was performed, were included in the study cohort of Sorok Island. (JONG HOON, L. 2020. Data Set - The Viral Respiratory Diseases of Sorok Island at Pandemic. In: OSF (ed.). Center for Open Science.)

- - 1. Complete blinded randomization

South Korea declared war against dementia in 2008 and prepared a National Dementia Plan every five years. The Korean National Assembly unanimously passed the Dementia Management Act' in 2011 and was enacted to diagnose and treat all Hansen subjects for Alzheimer's disease without exception(*5*). However, medical staff treated HD patients with VRD, while no one knew about dapson's relationship with viral inflammasomes. Therefore, all were in complete-blinded states. Participants were randomized in a 1:1 ratio to the DDS prescribed (+) group or the DDS unprescribed (-) control group. Randomization was unrestricted (no blocking or stratification), and the study's data team analysed the ICD codes in the EMR databases of Sorokdo National Hospital. According to the Dementia Management Act, the medical staff of Sorokdo National Hospital started a full investigation in 2011 for the treatment of dementia for all HD patients in Sorok Island. As a result, an anti-Alzheimer's disease drug was prescribed for Hansen subjects diagnosed with dementia, and doctors stopped prescribing dapsone for inactive HD patients. They have followed up all HD patients since 2011. The Dementia Management Act administered Dapsone to the trial group if we classified Dapsone unprescribed subjects as the control group.

A total of 9649 participants were enrolled, of whom 4685 were randomized to the dapsone prescription (DDS(+)) group and 4964 to the dapsone nonprescription (DDS(-)) group: 6983 in the viral respiratory disease (VRD)-diagnosed (+) group and 2666 in the undiagnosed (-) group from 2005 to 2020 on Sorok Island.

- 1. Analysis of Viral Respiratory Diseases Cohort
     1. Study Setting for ICD-10 code

Table S2. ICD-10 Study Code for Respiratory Diseases

| disease name | ICD-Code |
| --- | --- |
| Acute nasopharyngitis[common cold] | J00 |
| Acute sinusitis | **J01** |
| Other acute sinusitis, recurrent | J01.81 |
| Acute pharyngitis | **J02** |
| Acute pharyngitis, unspecified | J02.9 |
| Acute tonsillitis | **J03** |
| Streptococcal tonsillitis, recurrent | J03.01 |
| Acute tonsillitis, unspecified | J03.9 |
| Acute tonsillitis, unspecified, not specified as recurrent | J03.90 |
| Acute laryngitis | **J04.0** |
| Acute laryngopharyngitis | J06.0 |
| Acute upper respiratory infection, unspecified | J06.9 |
| Influenza due to identified zoonotic or pandemic influenza virus | **J09** |
| Influenza with other manifestations, seasonal influenza virus identified | J10.8 |
| Viral pneumonia, unspecified | **J12.9** |
| Bacterial pneumonia, NEC | **J15** |
| Other bacterial pneumonia | **J15.8** |
| Bacterial pneumonia, unspecified | **J15.9** |
| Pneumonia in bacterial diseases classified elsewhere | J17.0 |
| Pneumonia, organism unspecified | J18 |
| Bronchopneumonia, unspecified | J18.0 |
| Lobar pneumonia, unspecified | J18.1 |
| Other pneumonia, organism unspecified | **J18.8** |
| Pneumonia, unspecified | J18.9 |
| Acute bronchitis, unspecified | **J20.9** |
| Acute bronchiolitis | J21 |
| Acute bronchiolitis, unspecified | J21.9 |
| Unspecified acute lower respiratory infection | J22 |
| Vasomotor and allergic rhinitis | J30 |
| Vasomotor rhinitis | **J30.0** |
| Other seasonal allergic rhinitis | J30.2 |
| Other allergic rhinitis | J30.3 |
| Allergic rhinitis, unspecified | **J30.4** |
| Chronic rhinitis, nasopharyngitis and pharyngitis | J31 |
| Chronic rhinitis | **J31.0** |
| Chronic nasopharyngitis | J31.1 |
| Chronic pharyngitis | J31.2 |
| Chronic sinusitis | **J32** |
| Chronic maxillary sinusitis | J32.0 |
| Chronic pansinusitis | J32.4 |
| Other chronic sinusitis | J32.8 |
| Chronic sinusitis, unspecified | J32.9 |
| Abscess, furuncle and carbuncle of nose | **J34.0** |
| Deviated nasal septum | **J34.2** |
| Hypertrophy of nasal turbinates | J34.3 |
| Other specified disorders of nose and nasal sinuses | J34.8 |
| Chronic tonsillitis | J35.0 |
| Other chronic diseases of tonsils and adenoids | J35.8 |
| Peritonsillar abscess | J36 |
| Chronic laryngitis | J37.0 |
| Paralysis of vocal cords and larynx | **J38.0** |
| Other diseases of vocal cords | J38.3 |
| Oedema of larynx | J38.4 |
| Other diseases of larynx | J38.7 |
| Other diseases of upper respiratory tract | J39 |
| Retropharyngeal and parapharyngeal abscess | J39.0 |
| Other specified diseases of upper respiratory tract | J39.8 |
| Bronchitis, not specified as acute or chronic | J40 |
| Simple chronic bronchitis | J41.0 |
| Unspecified chronic bronchitis | J42 |
| Emphysema, unspecified | J43.9 |
| Other chronic obstructive pulmonary disease | **J44** |
| Chronic obstructive pulmonary disease with acute lower respiratory infection | J44.0 |
| Chronic obstructive pulmonary disease with acute exacerbation, unspecified | J44.1 |
| Other specified chronic obstructive pulmonary disease | J44.8 |
| Chronic obstructive pulmonary disease, unspecified | **J44.9** |
| Chronic obstructive pulmonary disease, unspecified, mild | J44.90 |
| Chronic obstructive pulmonary disease, unspecified, moderate | J44.91 |
| Chronic obstructive pulmonary disease, unspecified, unspecified | J44.99 |
| Asthma | J45 |
| Predominantly allergic asthma | J45.0 |
| Nonallergic asthma | J45.1 |
| Asthma, unspecified | J45.9 |
| Status asthmaticus | **J46** |
| Bronchiectasis | J47 |
| Pneumonitis due to food and vomit | J69.0 |
| Pulmonary oedema | J81 |
| Abscess of lung with pneumonia | J85.1 |
| Pleural effusion, NEC | J90 |
| Pneumothorax | J93 |
| Haemothorax | J94.2 |
| Chronic pulmonary insufficiency following surgery | J95.3 |
| Acute respiratory failure | J96.0 |
| Pulmonary collapse | J98.1 |
| Other specified respiratory disorders | J98.8 |
| Respiratory disorder, unspecified | J98.9 |

Table S2-1. ICD-10 Study Code for Viral Respiratory Diseases

| Study Code | description |
| --- | --- |
| J00 | Acute nasopharyngitis [common cold] |
| J01 | Acute sinusitis |
| J01.8 | Other acute sinusitis. Acute sinusitis involving more than one sinus but not pansinusitis |
| J02 | Acute pharyngitis |
| J02.9 | Acute pharyngitis, unspecified pharyngitis (acute):   - NOS - gangrenous - infective NOS - suppurative - ulcerative   Sore throat (acute) NOS |
| J03 | Acute tonsillitis |
| J03.9 | Acute tonsillitis, unspecified  Tonsillitis (acute):   - NOS - follicular - gangrenous - infective - ulcerative |
| J04.0 | Acute laryngitis  Laryngitis (acute):   - NOS - oedematous - subglottic - suppurative - ulcerative   Excl.: chronic laryngitis (J37.0)  influenzal laryngitis, influenza virus:   - identified (J09, J10.1) - not identified (J11.1) |
| J06.0 | Acute laryngopharyngitis |
| J06.9 | Acute upper respiratory infection, unspecified  Upper respiratory:   - disease, acute - infection NOS |
| J09 | Influenza due to identified zoonotic or pandemic influenza virus |
| J10.8 | Influenza with other manifestations, seasonal influenza virus identified. Encephalopathy due to influenza  Influenzal: seasonal influenza virus identified   - gastroenteritis - myocarditis (acute) |
| J12.9 | Viral pneumonia, unspecified |
| J20.9 | Acute bronchitis, unspecified |
| (https://icd.who.int/browse10/2019/en#/J20-J22)  'J00', 'J01', 'J01.81', 'J02', 'J02.9', 'J03', 'J03.9', 'J04.0', 'J06.0', 'J06.9', 'J09', 'J10.8', 'J12.9', 'J20.9' | |

Table S2-2. ICD-10 Study Code for Viral Respiratory Diseases

| disease name | ICD-Code | 2005 | 2006 | 2007 | 2008 | 2009 | 2010 | 2011 | 2012 | 2013 | 2014 | 2015 | 2016 | 2017 | 2018 | 2019 |
| --- | --- | --- | --- | --- | --- | --- | --- | --- | --- | --- | --- | --- | --- | --- | --- | --- |
| Acute nasopharyngitis[common cold] | J00 | 357 | 534 | 626 | 1134 | 1524 | 1541 | 1502 | 2080 | 2238 | 1870 | 1578 | 1240 | 926 | 732 | 286 |
| Acute sinusitis | **J01** | **1** |  |  | **42** | **42** | **72** | **171** | **217** | **284** | **161** | **151** | **118** | **109** | **31** |  |
| Other acute sinusitis, recurrent | J01.81 |  |  |  |  |  |  |  |  |  |  |  |  | 1 | 1 |  |
| Acute pharyngitis | **J02** | **23** | **23** | **25** | **233** | **244** | **270** | **263** | **436** | **564** | **485** | **471** | **384** | **339** | **163** | **75** |
| Acute pharyngitis, unspecified | J02.9 | 26 | 26 | 23 | 23 | 23 | 23 | 23 | 22 | 23 | 22 | 8 | 4 | 4 | 3 | 2 |
| Acute tonsillitis | **J03** | **2** | **6** | **32** | **65** | **68** | **70** | **70** | **117** | **179** | **92** | **62** | **33** | **19** | **11** | **1** |
| Streptococcal tonsillitis, recurrent | J03.01 |  |  |  |  |  |  |  |  |  |  |  | 1 | 1 | 1 | 1 |
| Acute tonsillitis, unspecified | J03.9 | 40 | 40 | 38 | 38 | 36 | 30 | 30 | 27 | 23 | 15 | 2 | 2 | 1 |  |  |
| Acute tonsillitis, unspecified, not specified as recurrent | J03.90 |  |  |  |  |  |  |  |  |  |  |  | 1 |  |  |  |
| Acute laryngitis | **J04.0** | **1** | **2** | **2** | **5** | **5** | **5** | **2** | **2** | **2** | **18** | **27** | **27** | **24** | **19** | **10** |
| Acute laryngopharyngitis | J06.0 |  |  |  | 2 | 2 | 2 | 2 | 2 | 2 | 2 | 2 | 2 | 1 | 1 | 1 |
| Acute upper respiratory infection, unspecified | J06.9 | 28 | 29 | 26 | 26 | 22 | 16 | 16 | **72** | **744** | **727** | **642** | **573** | **403** | **230** | **43** |
| Influenza due to identified zoonotic or pandemic influenza virus | **J09** |  |  |  |  |  |  |  |  | **267** | **252** | **205** | **161** | **112** | **74** | **32** |
| Influenza with other manifestations, seasonal influenza virus identified | J10.8 |  |  |  |  |  |  |  |  |  |  |  |  | 1 | 2 |  |
| Viral pneumonia, unspecified | **J12.9** |  |  |  |  |  |  |  |  | **33** | **33** | **33** | **33** | **34** | **33** |  |
| Bacterial pneumonia, NEC | **J15** |  |  | **1** | **18** | **24** | **18** | **143** | **17** | **6** | **2** |  |  |  |  |  |
| Other bacterial pneumonia | **J15.8** |  | **16** |  | **86** | **89** | **88** | **3** | **3** | **3** |  |  | **1** | **16** | **7** | **1** |
| Bacterial pneumonia, unspecified | **J15.9** | **5** | **5** | **5** | **95** | **5** | **6** | **136** | **258** | **638** | **218** | **68** | **32** | **5** | **4** | **4** |
| Pneumonia in bacterial diseases classified elsewhere | J17.0 |  | 20 | 20 | 20 | 20 | 20 | 20 | 20 | 20 | 20 |  |  |  |  |  |
| Pneumonia, organism unspecified | J18 |  |  |  |  |  |  |  |  |  | 1 | 1 |  |  |  |  |
| Bronchopneumonia, unspecified | J18.0 |  |  |  | 25 | 23 | 23 | 23 | 23 | 1 | 5 | 7 | 7 | 1 |  |  |
| Lobar pneumonia, unspecified | J18.1 |  |  |  |  |  |  |  | 12 |  |  |  |  |  |  |  |
| Other pneumonia, organism unspecified | **J18.8** |  | **17** |  | **111** | **111** | **110** | **110** | **110** | **127** | **17** | **17** | **17** |  |  |  |
| Pneumonia, unspecified | J18.9 | 36 | 36 | 36 | 36 | 15 | 7 | 7 | 7 | 7 | 9 | 16 | 23 | 18 | 13 | 8 |
| Acute bronchitis, unspecified | **J20.9** | **11** | **11** | **1** | **1** | **1** | **1** | **1** | **515** | **675** | **655** | **354** | **225** | **222** | **219** | **4** |
| Acute bronchiolitis | J21 |  |  | 4 | 4 | 4 |  |  |  |  |  |  |  |  |  |  |
| Acute bronchiolitis, unspecified | J21.9 | 1 | 1 |  |  |  |  |  |  |  |  |  |  |  |  |  |
| Unspecified acute lower respiratory infection | J22 | 1 | 1 | 1 |  | 1 | 1 | 1 | 1 | 1 |  |  |  |  |  |  |
| Vasomotor and allergic rhinitis | J30 |  |  |  |  |  |  |  |  |  | 4 | 6 | 6 | 6 | 5 | 1 |
| Vasomotor rhinitis | **J30.0** |  |  | **3** | **45** | **54** | **96** | **99** | **182** | **180** | **110** | **96** | **28** | **30** | **29** | **9** |
| Other seasonal allergic rhinitis | J30.2 |  |  |  |  |  |  |  |  |  | 2 | 3 | 4 | 3 | 2 | 1 |
| Other allergic rhinitis | J30.3 |  | 13 | 13 | 13 | 13 | 13 | 13 | 13 | 13 | 36 | 54 | 49 | 42 | 33 | 9 |
| Allergic rhinitis, unspecified | **J30.4** | **11** | **11** | **60** | **189** | **205** | **197** | **151** | **142** | **178** | **129** | **102** | **71** | **47** | **40** | **21** |
| Chronic rhinitis, nasopharyngitis and pharyngitis | J31 |  |  |  |  |  |  |  |  | 2 | 2 | 2 | 1 | 1 | 1 |  |
| Chronic rhinitis | **J31.0** | **10** | **40** | **40** | **16** | **95** | **91** | **64** | **19** | **15** | **18** | **19** | **16** | **14** | **11** | **1** |
| Chronic nasopharyngitis | J31.1 |  |  |  |  |  |  |  |  | 2 | 17 | 33 | 49 | 38 | 30 | 8 |
| Chronic pharyngitis | J31.2 |  | 27 |  |  |  |  |  |  | 79 | 168 | 226 |  | 188 | 130 |  |
| Chronic sinusitis | **J32** |  |  |  | **571** | **636** | **567** | **455** | **345** | **291** | **155** | **128** |  | **106** |  | **5** |
| Chronic maxillary sinusitis | J32.0 |  |  |  |  |  |  |  |  | 1 | 9 | 20 |  | 17 |  | 5 |
| Chronic pansinusitis | J32.4 |  |  |  |  |  |  |  |  |  |  | 2 | 2 | 4 | 4 | 4 |
| Other chronic sinusitis | J32.8 |  |  |  |  |  |  |  |  |  |  |  | 1 | 1 | 1 | 1 |
| Chronic sinusitis, unspecified | J32.9 | 11 | 19 | 18 | 16 | 15 | 15 | **44** | **41** | 42 | 33 | 32 | 32 | 29 |  |  |
| Abscess, furuncle and carbuncle of nose | **J34.0** |  | **1** | **1** | **122** | **127** | **59** | **59** | **43** | **24** | **51** | **47** | **47** | **41** | **25** | **16** |
| Deviated nasal septum | **J34.2** |  |  |  | **5** | **56** | **56** | **56** | **120** | **70** | **51** | **65** | **22** | **26** | **27** | **6** |
| Hypertrophy of nasal turbinates | J34.3 | 1 | 1 | 1 | 1 | 1 | 1 |  |  |  |  |  |  |  |  |  |
| Other specified disorders of nose and nasal sinuses | J34.8 | 2 | 4 |  | 4 | 4 | 4 | 3 | 3 | 5 | 5 | 7 | 6 | 2 | 2 | 2 |
| Chronic tonsillitis | J35.0 |  |  |  |  |  |  |  |  |  | 5 | 10 | 12 | 7 | 4 | 2 |
| Other chronic diseases of tonsils and adenoids | J35.8 |  |  |  |  |  |  |  | 5 | 5 |  |  |  |  |  |  |
| Peritonsillar abscess | J36 |  |  |  |  |  |  |  | 7 | 7 | 7 | 7 | 7 | 7 |  |  |
| Chronic laryngitis | J37.0 | 6 | 6 | 6 |  | 7 | 6 | 6 | 6 | 5 | 7 | 12 | 15 | 11 | 8 | 3 |
| Paralysis of vocal cords and larynx | **J38.0** | **1** | **1** | **1** |  | **16** | **16** | **14** | **14** | **14** | **14** | **14** | **14** | **10** | **10** |  |
| Other diseases of vocal cords | J38.3 |  |  |  |  |  |  |  | 9 | 9 |  |  |  |  |  |  |
| Oedema of larynx | J38.4 |  |  |  |  |  |  | 1 | 1 | 1 | 1 | 1 |  |  |  |  |
| Other diseases of larynx | J38.7 |  |  |  |  |  |  |  |  |  |  | 1 | 1 | 1 | 1 |  |
| Other diseases of upper respiratory tract | J39 |  |  |  | 1 |  |  |  |  |  |  |  |  |  |  |  |
| Retropharyngeal and parapharyngeal abscess | J39.0 |  |  |  |  |  |  |  | 16 | 16 | 16 |  |  |  |  |  |
| Other specified diseases of upper respiratory tract | J39.8 |  |  |  |  |  |  |  |  |  |  |  |  |  | 1 | 1 |
| Bronchitis, not specified as acute or chronic | J40 | 4 | 4 | 4 | 4 | 4 | 4 | 4 | 4 | 1 | 1 |  |  |  |  |  |
| Simple chronic bronchitis | J41.0 |  |  |  |  |  |  |  |  | 2 | 3 | 5 | 5 | 2 | 1 | 1 |
| Unspecified chronic bronchitis | J42 | 11 | 58 | 28 | 41 | 41 | 41 | 30 | 13 | 13 |  |  |  |  |  |  |
| Emphysema, unspecified | J43.9 | 1 | 1 | 1 | 1 |  |  |  |  |  |  |  |  |  |  |  |
| Other chronic obstructive pulmonary disease | **J44** |  |  |  | **65** | **64** | **64** | **64** |  |  |  |  |  |  |  |  |
| Chronic obstructive pulmonary disease with acute lower respiratory infection | J44.0 |  |  |  |  |  |  |  |  |  | 2 | 2 |  | 2 | 1 |  |
| Chronic obstructive pulmonary disease with acute exacerbation, unspecified | J44.1 |  |  |  |  |  |  |  |  |  | 1 | 1 | 1 | 1 | 1 | 1 |
| Other specified chronic obstructive pulmonary disease | J44.8 |  |  |  | 1 |  |  |  |  |  |  |  |  |  |  |  |
| Chronic obstructive pulmonary disease, unspecified | **J44.9** | **35** | **29** | **144** | **242** | **356** | **404** | **382** | **1257** | **1447** | **948** | **631** | **473** | **375** | **212** |  |
| Chronic obstructive pulmonary disease, unspecified, mild | J44.90 |  |  |  |  |  |  |  |  |  |  |  |  | 1 | 1 |  |
| Chronic obstructive pulmonary disease, unspecified, moderate | J44.91 |  |  |  |  |  |  |  |  |  |  |  |  | 10 | 6 | 5 |
| Chronic obstructive pulmonary disease, unspecified, unspecified | J44.99 |  |  |  |  |  |  |  |  |  |  |  |  | 1 |  |  |
| Asthma | J45 | 6 | 5 | 5 | 5 | 5 | 5 | 5 | 4 | 2 | 3 | 2 | 1 | 1 | 1 |  |
| Predominantly allergic asthma | J45.0 |  |  | 40 | 40 |  |  |  |  |  |  |  |  |  |  |  |
| Nonallergic asthma | J45.1 |  |  | 36 |  |  |  |  |  |  |  |  |  |  |  |  |
| Asthma, unspecified | J45.9 |  |  |  |  |  |  |  |  | 5 | 6 | 7 | 6 | 5 | 3 | 2 |
| Status asthmaticus | **J46** |  |  |  | **4** | **4** | **4** |  |  |  |  |  |  |  |  |  |
| Bronchiectasis | J47 | 2 | 2 | 2 | 2 | 2 | 2 | 2 | 2 | 3 | 3 | 2 | 2 | 2 |  |  |
| Pneumonitis due to food and vomit | J69.0 |  | 5 | 5 | 6 | 6 | 1 | **9** | **61** | **89** | 34 | 39 | 36 | 35 | 34 | 1 |
| Pulmonary oedema | J81 |  |  |  |  |  |  |  |  |  |  | 3 | 2 | 2 | 2 | 1 |
| Abscess of lung with pneumonia | J85.1 |  | 5 | 5 | 5 |  |  |  |  |  |  |  |  |  |  |  |
| Pleural effusion, NEC | J90 |  |  |  |  |  |  |  |  |  |  | 2 | 1 | 2 |  |  |
| Pneumothorax | J93 | 1 | 5 | 1 | 1 | 1 | 1 | 1 | 1 | 1 | 1 | 1 |  |  |  |  |
| Haemothorax | J94.2 |  |  |  |  |  |  |  |  |  | 2 | 1 | 1 |  |  |  |
| Chronic pulmonary insufficiency following surgery | J95.3 |  |  |  |  |  |  |  |  |  |  | 1 |  | 1 |  |  |
| Acute respiratory failure | J96.0 |  |  |  | 20 |  |  |  |  |  |  |  |  |  |  |  |
| Pulmonary collapse | J98.1 |  |  |  |  |  |  |  |  |  |  | 1 |  |  |  |  |
| Other specified respiratory disorders | J98.8 |  |  |  |  |  |  |  |  |  |  |  |  | 1 |  |  |
| Respiratory disorder, unspecified | J98.9 |  |  |  |  |  |  |  |  |  |  |  |  | 5 | 2 | 1 |

- - 1. Statistical framework

We used the software programs Object-Relational DBMS and Google spreadsheet with SPSS. T tests, the Mann–Whitney U test, one-way repeated-measures ANOVA and post hoc Tukey’s honestly significant difference (HSD) test were applied. A significant T test was performed among each group of T1: DDS(+)/VRD(+), T2: DDS(-)/VRD(+), T3: DDS(+)/VRD(-), T4: DDS(-)/VRD(-).

Section 4. Primary T test

4.1. Primary T test for T1:T2:T3:T4

Table S4. Primary T1:T2:T3:T4 study for the retrospective cohort study at Sorok Island.

| year | T1^*^ | T2^*^ | T3^*^ | T4^*^ | Sum | Mean | SD^f^ | 95% CI^g^ | [CI^g^ | CI^g^] | χ2^*^ | p value |
| --- | --- | --- | --- | --- | --- | --- | --- | --- | --- | --- | --- | --- |
| 2005 | 148 | 95 | 233 | 268 | 744 | 186 | 78.86 | 11.41 | 174.59 | 264.86 | 13.5772 | 0.000229 |
| 2006 | 166 | 111 | 237 | 208 | 722 | 180.5 | 54.74 | 8.05 | 172.45 | 235.24 | 3.0793 | 0.079295 |
| 2007 | 170 | 115 | 252 | 185 | 722 | 180.5 | 56.37 | 8.29 | 172.21 | 236.87 | 0.2794 | 0.597118 |
| 2008 | 207 | 128 | 219 | 148 | 702 | 175.5 | 44.34 | 6.61 | 168.89 | 219.84 | 0.3293 | 0.566073 |
| 2009 | 222 | 135 | 196 | 115 | 668 | 167 | 50.31 | 7.69 | 159.31 | 217.31 | 0.0498 | 0.823418 |
| 2010 | 202 | 170 | 186 | 105 | 663 | 165.75 | 42.55 | 6.54 | 159.21 | 208.30 | 6.2203 | 0.012629 |
| 2011 | 205 | 164 | 170 | 117 | 656 | 164 | 36.18 | 5.58 | 158.42 | 200.18 | 0.8918 | 0.344987 |
| 2012 | 237 | 211 | 103 | 102 | 653 | 163.25 | 70.95 | 10.97 | 152.28 | 234.20 | 0.3981 | 0.528071 |
| 2013 | 269 | 349 | 8 | 23 | 649 | 162.25 | 172.68 | 26.79 | 135.46 | 334.93 | 3.7892 | 0.051583 |
| 2014 | 236 | 349 | 6 | 32 | 623 | 155.75 | 164.85 | 26.16 | 129.59 | 320.60 | 9.0547 | 0.00262 |
| 2015 | 227 | 325 | 7 | 44 | 603 | 150.75 | 150.82 | 24.33 | 126.42 | 301.57 | 14.7575 | 0.000122 |
| 2016 | 207 | 319 | 4 | 61 | 591 | 147.75 | 142.63 | 23.25 | 124.50 | 290.38 | 27.7773 | < 0.00001^e^ |
| 2017 | 193 | 301 | 14 | 55 | 563 | 140.75 | 131.44 | 21.96 | 118.79 | 272.19 | 9.1835 | 0.002442 |
| 2018 | 178 | 303 | 15 | 60 | 556 | 139 | 129.14 | 21.66 | 117.34 | 268.14 | 8.2801 | 0.004008 |
| 2019 | 155 | 297 | 13 | 69 | 534 | 133.5 | 123.66 | 21.21 | 112.29 | 257.16 | 10.9433 | 0.000939 |
| Sum | 3022 | 3372 | 1663 | 1592 |  |  |  |  |  |  |  |  |
| Mean | 201.47 | 224.80 | 110.87 | 106.13 |  |  |  |  |  |  |  |  |
| SD | 33.86 | 97.50 | 103.80 | 70.30 |  |  |  |  |  |  |  |  |
| 95% CI | 1.21 | 3.29 | 4.99 | 3.46 |  |  |  |  |  |  |  |  |
| [ | 200.26 | 221.51 | 105.87 | 102.68 |  |  |  |  |  |  |  |  |
| ] | 202.67 | 228.09 | 115.86 | 109.59 |  |  |  |  |  |  |  |  |
| The chi-square is 281.826. | | | | | |  |  |  |  |  |  |  |
| The p value is < 0.00001. I t is significant at p < .05. | | | | | | | | | | | | |
| Blue means we are dealing with dependent variables; red, independent. | | | | | | | | | | | | |

### Table S4-1. One-way ANOVA Calculator for Independent Measures of Primary T1:T2:T3:T4 study for the retrospective cohort study at Sorok Island

| \|  \| T1 \| T2 \| T3 \| T4 \| Total \| \| --- \| --- \| --- \| --- \| --- \| --- \| \| N \| 15 \| 15 \| 15 \| 15 \| 60 \| \| ∑X \| 3022 \| 3372 \| 1663 \| 1592 \| 9649 \| \| Mean \| 201.4667 \| 224.8 \| 110.8667 \| 106.1333 \| 160.817 \| \| ∑X^2^ \| 624884 \| 891104 \| 335199 \| 238156 \| 2089343 \| \| Std.Dev. \| 33.8608 \| 97.4967 \| 103.795 \| 70.3013 \| 95.4581 \|  \| *Source* \| *SS* \| *df* \| *MS* \|  \| \| --- \| --- \| --- \| --- \| --- \| \| Between-treatments \| 168473.3833 \| 3 \| 56157.7944 \| *F* = 8.51914 \| \| Within-treatments \| 369149.6 \| 56 \| 6591.9571 \|  \| \| Total \| 537622.9833 \| 59 \|  \|  \|   The *f*-ratio value is 8.51914. The *p* value is .000094. The result is significant at *p* < .05. |
| --- | --- | --- | --- | --- | --- | --- | --- | --- | --- | --- | --- | --- | --- | --- | --- | --- | --- | --- | --- | --- | --- | --- | --- | --- | --- | --- | --- | --- | --- | --- | --- | --- | --- | --- | --- | --- | --- | --- | --- | --- | --- | --- | --- | --- | --- | --- | --- | --- | --- | --- | --- | --- | --- | --- | --- | --- |

### Table S4-2. Post hoc Tukey HSD

| *Pairwise Comparisons* | | HSD_.05_ = 78.5016 HSD_.01_ = 96.6119 | Q_.05_ = 3.7447    Q_.01_ = 4.6086 |
| --- | --- | --- | --- |
| T_1_:T_2_ | M_1_ = 201.47 M_2_ = 224.80 | 23.33 | Q = 1.11 (*p* = .86003) |
| T_1_:T_3_ | M_1_ = 201.47 M_3_ = 110.87 | 90.60 | Q = 4.32 (*p* = .01755) |
| T_1_:T_4_ | M_1_ = 201.47 M_4_ = 106.13 | 95.33 | Q = 4.55 (*p* = .01130) |
| T_2_:T_3_ | M_2_ = 224.80 M_3_ = 110.87 | 113.93 | Q = 5.43 (*p* = .00173) |
| T_2_:T_4_ | M_2_ = 224.80 M_4_ = 106.13 | 118.67 | Q = 5.66 (*p* = .00104) |
| T_3_:T_4_ | M_3_ = 110.87 M_4_ = 106.13 | 4.73 | Q = 0.23 (*p* = .99853) |

We excluded the T1:T2 and T3:T4 tests.

### Table S4-3. One-way ANOVA Calculator for repeated Measures of Primary T1:T2:T3:T4 study for the retrospective cohort study at Sorok Island

| \|  \| T1 \| T2 \| T3 \| T4 \| Total \| \| --- \| --- \| --- \| --- \| --- \| --- \| \| N \| 15 \| 15 \| 15 \| 15 \| 60 \| \| ∑X \| 3022 \| 3372 \| 1663 \| 1592 \| 9649 \| \| Mean \| 201.4667 \| 224.8 \| 110.8667 \| 106.1333 \| 160.817 \| \| ∑X^2^ \| 624884 \| 891104 \| 335199 \| 238156 \| 2089343 \| \| Std.Dev. \| 33.8608 \| 97.4967 \| 103.795 \| 70.3013 \| 95.4581 \|  \| ***Source*** \| ***SS*** \| ***df*** \| ***MS*** \|  \| \| --- \| --- \| --- \| --- \| --- \| \| Between-treatments \| 168473.3833 \| 3 \| 56157.7944 \| *F* = 6.65081 \| \| Within-treatments \| 369149.6 \| 56 \| 6591.9571 \|  \| \| Total \| 354637.8667 \| 42 \| 8443.7587 \|  \|   The *F*-ratio value is 6.65081. The *p* value is .000891. The result is significant at *p* < .05. |
| --- | --- | --- | --- | --- | --- | --- | --- | --- | --- | --- | --- | --- | --- | --- | --- | --- | --- | --- | --- | --- | --- | --- | --- | --- | --- | --- | --- | --- | --- | --- | --- | --- | --- | --- | --- | --- | --- | --- | --- | --- | --- | --- | --- | --- | --- | --- | --- | --- | --- | --- | --- | --- | --- | --- | --- | --- |

### Fig. S2. Study Results of Primary T1:T2:T3:T4


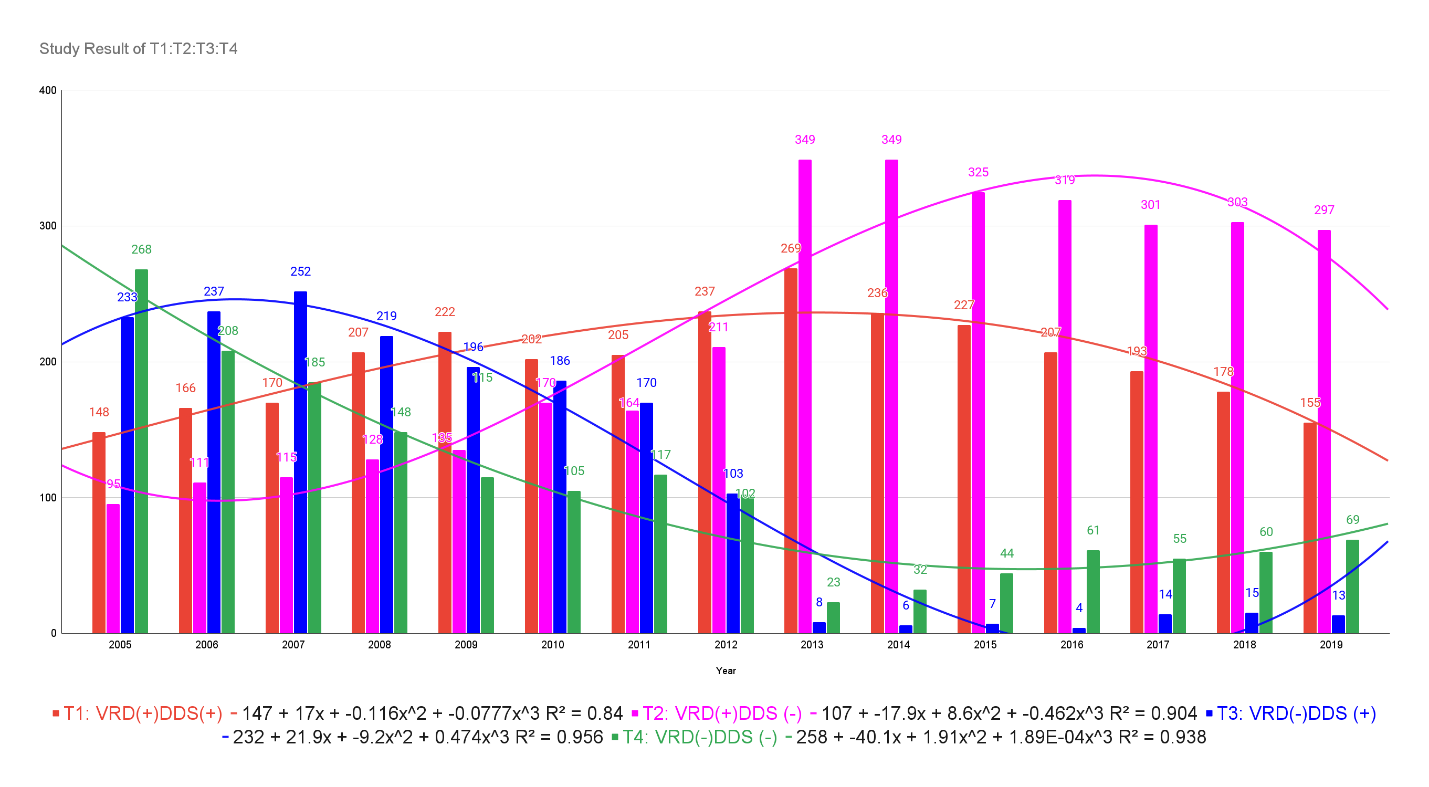


4.2. T test for Primary T1:T3

Table S4-4. Primary T test for T1:T3

| Independent Means | Dependent Means |
| --- | --- |
| Significance Level: 0.05, Two-tailed hypothesis. | Significance Level: 0.05, Two-tailed hypothesis. |
| The t value is 3.21393. The p value is .003287. The result is significant at p < .05. | The value of t is -2.896042. The value of p is .01173. The result is significant at p < .05. |
| Difference Scores Calculations  Treatment 1  N1: 15  df1 = N - 1 = 15 - 1 = 14  M1: 201.47  SS1: 16051.73  s21 = SS1/(N - 1) = 16051.73/(15-1) = 1146.55  Treatment 2  N2: 15  df2 = N - 1 = 15 - 1 = 14  M2: 110.87  SS2: 150827.73  s22 = SS2/(N - 1) = 150827.73/(15-1) = 10773.41  T value Calculation  s2p = ((df1/(df1 + df2)) * s21) + ((df2/(df2 + df2)) * s22) = ((14/28) * 1146.55) + ((14/28) * 10773.41) = 5959.98  s2M1 = s2p/N1 = 5959.98/15 = 397.33  s2M2 = s2p/N2 = 5959.98/15 = 397.33  t = (M1 - M2)/√(s2M1 + s2M2) = 90.6/√794.66 = 3.21 | Difference Scores Calculations  Mean: -90.6  μ = 0  S2 = SS⁄df = 205525.6/(15-1) = 14680.4  S2 M = S2/N = 14680.4/15 = 978.69  SM = √S2 M = √978.69 = 31.28  T value Calculation  t = (M - μ)/SM = (-90.6 - 0)/31.28 = -2.9 |

Table S4-5. Effect Size Calculator for T Test for T1:T3

| T1  Mean (M):201.47  Standard deviation (s):16051.73  Sample size (n):15 | T3  Mean (M):110.87  Standard deviation (s):150827.7  Sample size (n):15 |
| --- | --- |
| Cohen's d = (110.87 - 201.47) ⁄ 107253.562 = 0.000845.  Glass's delta = (110.87 - 201.47) ⁄ 16051.73 = 0.005644.  Hedges' g = (110.87 - 201.47) ⁄ 107253.562 = 0.000845. | |

Fig. S3. Primary Study Result of T1:T3


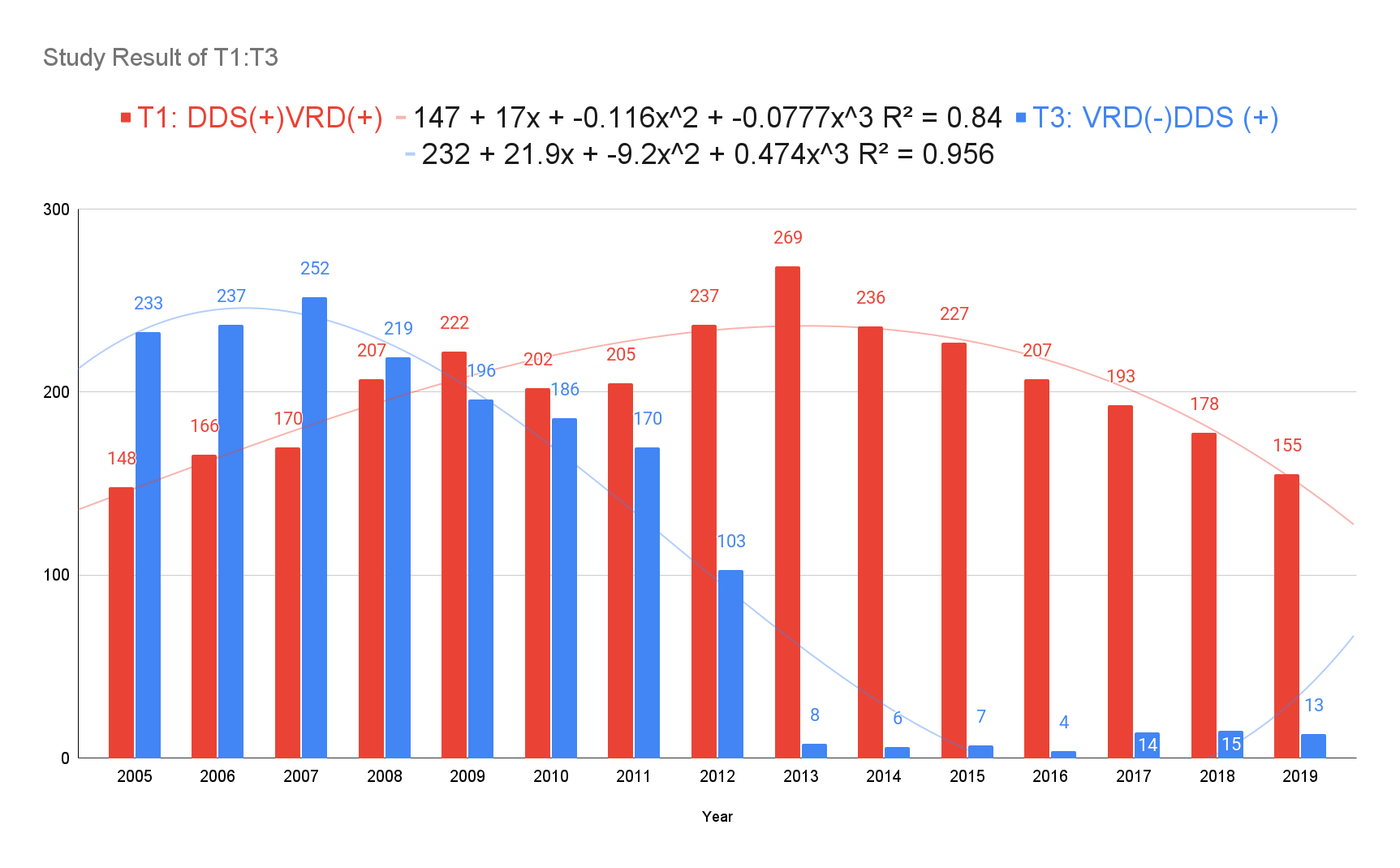


4.3. T test for Primary T1:T4

Table S4-6. Primary T test for T1:T4

| Independent Means | Dependent Means |
| --- | --- |
| Significance Level: 0.05, Two-tailed hypothesis. | Significance Level: 0.05, Two-tailed hypothesis. |
| The t value is 4.73177. The p value is .000058. The result is significant at p < .05. | The value of t is -3.866408. The value of p is .00171. The result is significant at p < .05. |
| Difference Scores Calculations  Treatment 1  N1: 15  df1 = N - 1 = 15 - 1 = 14  M1: 201.47  SS1: 16051.73  s21 = SS1/(N - 1) = 16051.73/(15-1) = 1146.55  Treatment 2  N2: 15  df2 = N - 1 = 15 - 1 = 14  M2: 106.13  SS2: 69191.73  s22 = SS2/(N - 1) = 69191.73/(15-1) = 4942.27  T value Calculation  s2p = ((df1/(df1 + df2)) * s21) + ((df2/(df2 + df2)) * s22) = ((14/28) * 1146.55) + ((14/28) * 4942.27) = 3044.41  s2M1 = s2p/N1 = 3044.41/15 = 202.96  s2M2 = s2p/N2 = 3044.41/15 = 202.96  t = (M1 - M2)/√(s2M1 + s2M2) = 95.33/√405.92 = 4.73 | Difference Scores Calculations  Mean: -95.33  μ = 0  S2 = SS⁄df = 127671.33/(15-1) = 9119.38  S2 M = S2/N = 9119.38/15 = 607.96  SM = √S2 M = √607.96 = 24.66  T value Calculation  t = (M - μ)/SM = (-95.33 - 0)/24.66 = -3.87 |

Table S4-7. Effect Size Calculator for T Test for T1:T4

| T1  Mean (M):201.47  Standard deviation (s):16051.73  Sample size (n):15 | T4  Mean (M):106.13  Standard deviation (s):69191.73  Sample size (n):15 |
| --- | --- |
| Cohen's d = (106.13 - 201.47) ⁄ 50225.26026 = 0.001898.  Glass's delta = (106.13 - 201.47) ⁄ 16051.73 = 0.00594.  Hedges' g = (106.13 - 201.47) ⁄ 50225.26026 = 0.001898. | |

Fig. S4. Primary Study Results of T1:T4


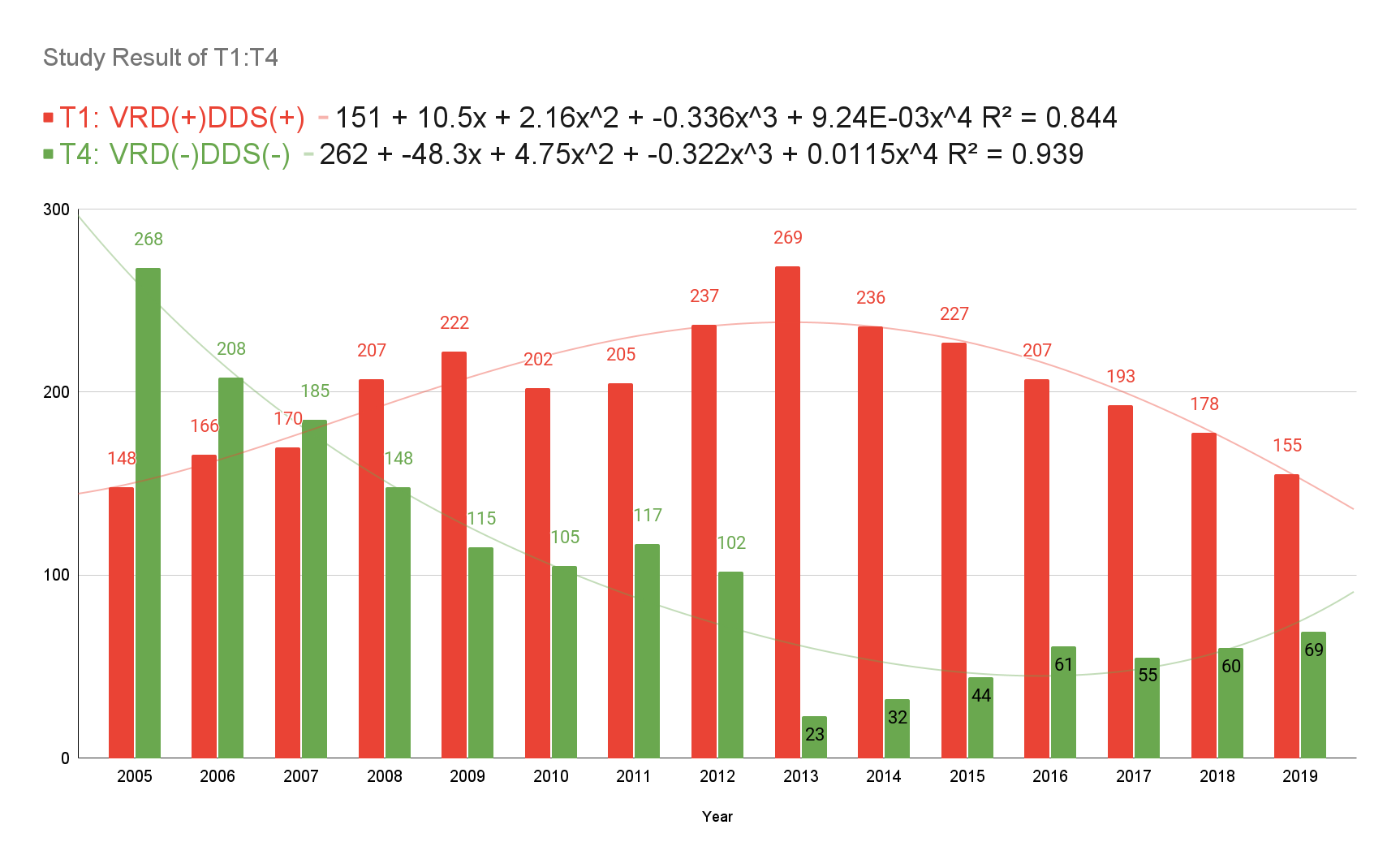


4.4. T test for Primary T2:T3

Table S4-8. Primary T test for T2:T3

| Independent Means | Dependent Means |
| --- | --- |
| Significance Level: 0.05, Two-tailed hypothesis. | Significance Level: 0.05, Two-tailed hypothesis. |
| The t value is 3.09865. The p value is .004395. The result is significant at p < .05. | The value of t is -2.200066. The value of p is .04509. The result is significant at p < .05. |
| Difference Scores Calculations  Treatment 1  N1: 15  df1 = N - 1 = 15 - 1 = 14  M1: 224.8  SS1: 133078.4  s21 = SS1/(N - 1) = 133078.4/(15-1) = 9505.6  Treatment 2  N2: 15  df2 = N - 1 = 15 - 1 = 14  M2: 110.87  SS2: 150827.73  s22 = SS2/(N - 1) = 150827.73/(15-1) = 10773.41  T value Calculation  s2p = ((df1/(df1 + df2)) * s21) + ((df2/(df2 + df2)) * s22) = ((14/28) * 9505.6) + ((14/28) * 10773.41) = 10139.5  s2M1 = s2p/N1 = 10139.5/15 = 675.97  s2M2 = s2p/N2 = 10139.5/15 = 675.97  t = (M1 - M2)/√(s2M1 + s2M2) = 113.93/√1351.93 = 3.1 | Difference Scores Calculations  Mean: -113.93  μ = 0  S2 = SS⁄df = 563182.93/(15-1) = 40227.35  S2 M = S2/N = 40227.35/15 = 2681.82  SM = √S2 M = √2681.82 = 51.79  T value Calculation  t = (M - μ)/SM = (-113.93 - 0)/51.79 = -2.2 |

Table S4-9. Effect Size Calculator for T Test for T2:T3

| T2  Mean (M):224.8  Standard deviation (s):133078.4  Sample size (n):15 | T3  Mean (M):110.87  Standard deviation (s):150827.7  Sample size (n):15 |
| --- | --- |
| Cohen's d = (110.87 - 224.8) ⁄ 142230.193057 = 0.000801.  Glass's delta = (110.87 - 224.8) ⁄ 133078.4 = 0.000856.  Hedges' g = (110.87 - 224.8) ⁄ 142230.193057 = 0.000801. | |

Fig. S5. Primary Study Results of T2:T3


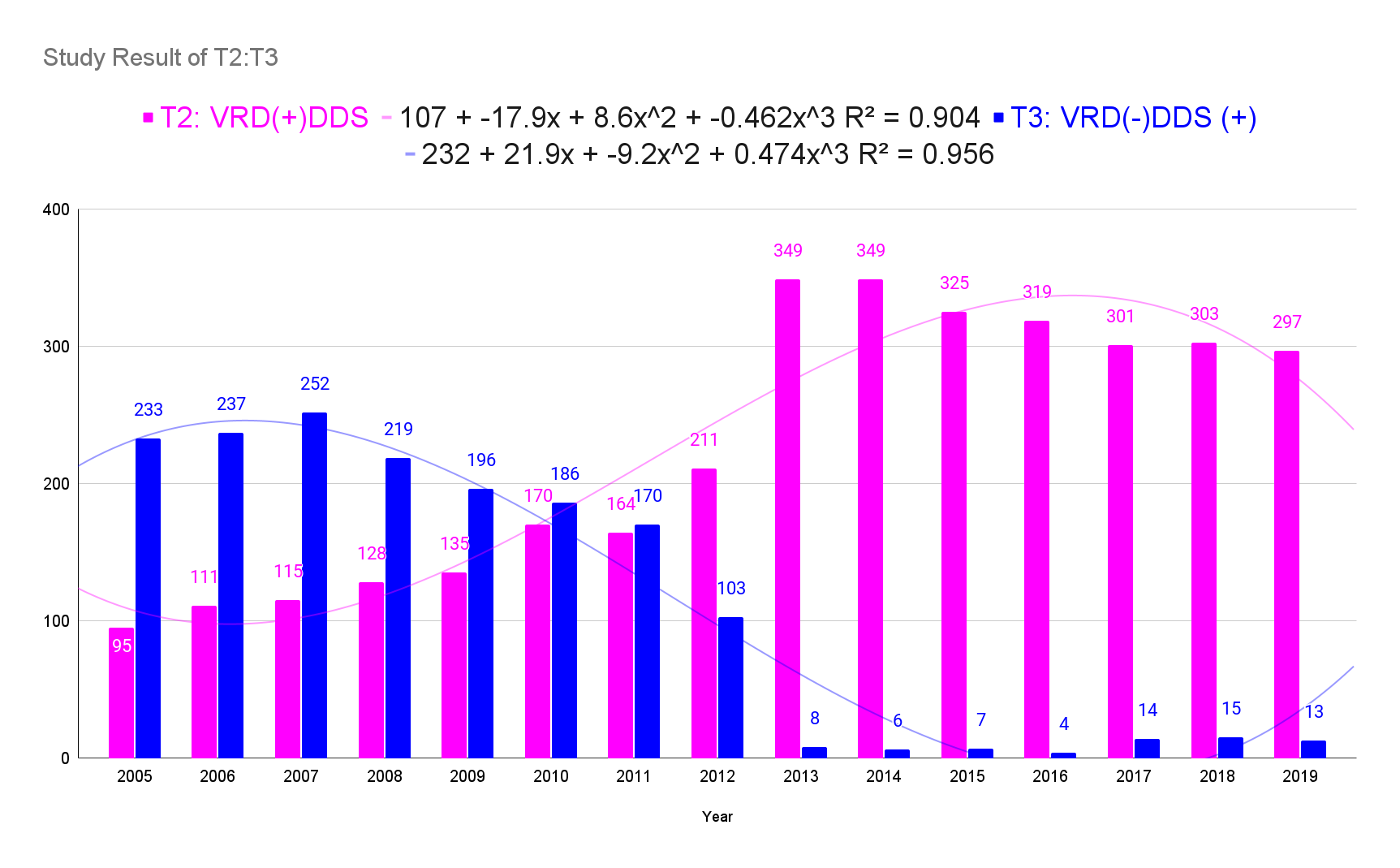


4.5. T test for Primary T2:T4

Table S4-10. Primary T test for T2:T4

| Independent Means | Dependent Means |
| --- | --- |
| Significance Level: 0.05, Two-tailed hypothesis. | Significance Level: 0.05, Two-tailed hypothesis. |
| The t value is 3.8236. The p value is .000673. The result is significant at p < .05. | The value of t is -2.804558. The value of p is .01405. The result is significant at p < .05. |
| Difference Scores Calculations  Treatment 1  N1: 15  df1 = N - 1 = 15 - 1 = 14  M1: 224.8  SS1: 133078.4  s21 = SS1/(N - 1) = 133078.4/(15-1) = 9505.6  Treatment 2  N2: 15  df2 = N - 1 = 15 - 1 = 14  M2: 106.13  SS2: 69191.73  s22 = SS2/(N - 1) = 69191.73/(15-1) = 4942.27  T value Calculation  s2p = ((df1/(df1 + df2)) * s21) + ((df2/(df2 + df2)) * s22) = ((14/28) * 9505.6) + ((14/28) * 4942.27) = 7223.93  s2M1 = s2p/N1 = 7223.93/15 = 481.6  s2M2 = s2p/N2 = 7223.93/15 = 481.6  t = (M1 - M2)/√(s2M1 + s2M2) = 118.67/√963.19 = 3.82 | Difference Scores Calculations  Mean: -118.67  μ = 0  S2 = SS⁄df = 375965.33/(15-1) = 26854.67  S2 M = S2/N = 26854.67/15 = 1790.31  SM = √S2 M = √1790.31 = 42.31  T value Calculation  t = (M - μ)/SM = (-118.67 - 0)/42.31 = -2.8 |

Table S4-11. Effect Size Calculator for T Test for T2:T4

| T2  Mean (M):224.8  Standard deviation (s):133078.4  Sample size (n):15 | T4  Mean (M):106.13  Standard deviation (s):69191.73  Sample size (n):15 |
| --- | --- |
| Cohen's d = (106.13 - 224.8) ⁄ 106059.785138 = 0.001119.  Glass's delta = (106.13 - 224.8) ⁄ 133078.4 = 0.000892.  Hedges' g = (106.13 - 224.8) ⁄ 106059.785138 = 0.001119. | |

Fig. S6. Primary Study Results of T2:T4


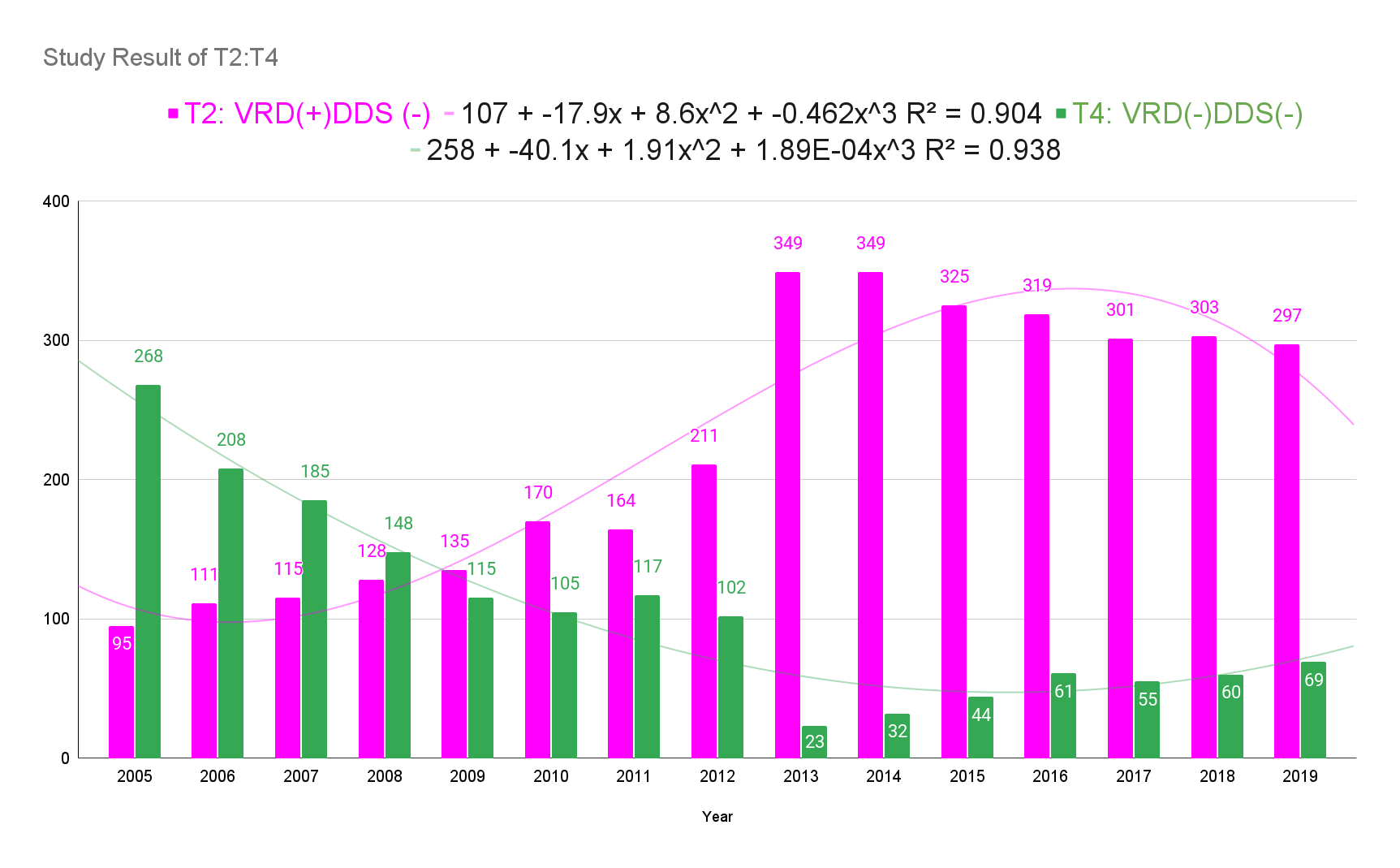


**Section 5. Statistical Renormalization Analysis for the cohort study on Sorok Island**

Table S5-1. Renormalization Study for Respiratory Diseases in Sorok Island

| disease name | ICD-Code | 2005 | 2006 | 2007 | 2008 | 2009 | 2010 | 2011 | 2012 | 2013 | 2014 | 2015 | 2016 | 2017 | 2018 | 2019 |
| --- | --- | --- | --- | --- | --- | --- | --- | --- | --- | --- | --- | --- | --- | --- | --- | --- |
| Bacterial pneumonia, NEC | J15 |  |  | 1 | 18 | 24 | 18 | 143 | 17 | 6 | 2 |  |  |  |  |  |
| Other bacterial pneumonia | J15.8 |  | 16 |  | 86 | 89 | 88 | 3 | 3 | 3 |  |  | 1 | 16 | 7 | 1 |
| Bacterial pneumonia, unspecified | J15.9 | 5 | 5 | 5 | 95 | 5 | 6 | 136 | 258 | 638 | 218 | 68 | 32 | 5 | 4 | 4 |
| Pneumonia in bacterial diseases classified elsewhere | J17.0 |  | 20 | 20 | 20 | 20 | 20 | 20 | 20 | 20 | 20 |  |  |  |  |  |
| Bronchopneumonia, unspecified | J18.0 |  |  |  | 25 | 23 | 23 | 23 | 23 | 1 | 5 | 7 | 7 | 1 |  |  |
| Lobar pneumonia, unspecified | J18.1 |  |  |  |  |  |  |  | 12 |  |  |  |  |  |  |  |
| Other pneumonia, organism unspecified | J18.8 |  | 17 |  | 111 | 111 | 110 | 110 | 110 | 127 | 17 | 17 | 17 |  |  |  |
| Pneumonia, unspecified | J18.9 | 36 | 36 | 36 | 36 | 15 | 7 | 7 | 7 | 7 | 9 | 16 | 23 | 18 | 13 | 8 |
| Acute bronchitis, unspecified | **J20.9** | **11** | **11** | **1** | **1** | **1** | **1** | **1** | **515** | **675** | **655** | **354** | **225** | **222** | **219** | **4** |
| Acute bronchiolitis | J21 |  |  | 4 | 4 | 4 |  |  |  |  |  |  |  |  |  |  |
| Acute bronchiolitis, unspecified | J21.9 | 1 | 1 |  |  |  |  |  |  |  |  |  |  |  |  |  |
| Vasomotor rhinitis | **J30.0** |  |  | **3** | **45** | **54** | **96** | **99** | **182** | **180** | **110** | **96** | **28** | **30** | **29** | **9** |
| Other allergic rhinitis | J30.3 |  | 13 | 13 | 13 | 13 | 13 | 13 | 13 | 13 | 36 | 54 | 49 | 42 | 33 | 9 |
| Allergic rhinitis, unspecified | **J30.4** | **11** | **11** | **60** | **189** | **205** | **197** | **151** | **142** | **178** | **129** | **102** | **71** | **47** | **40** | **21** |
| Chronic rhinitis | **J31.0** | **10** | **40** | **40** | **16** | **95** | **91** | **64** | **19** | **15** | **18** | **19** | **16** | **14** | **11** | **1** |
| Chronic nasopharyngitis | J31.1 |  |  |  |  |  |  |  |  | 2 | 17 | 33 | 49 | 38 | 30 | 8 |
| Chronic pharyngitis | J31.2 |  | 27 |  |  |  |  |  |  | 79 | 168 | 226 |  | 188 | 130 |  |
| Chronic sinusitis | **J32** |  |  |  | **571** | **636** | **567** | **455** | **345** | **291** | **155** | **128** |  | **106** |  | **5** |
| Chronic maxillary sinusitis | J32.0 |  |  |  |  |  |  |  |  | 1 | 9 | 20 |  | 17 |  | 5 |
| Chronic pansinusitis | J32.4 |  |  |  |  |  |  |  |  |  |  | 2 | 2 | 4 | 4 | 4 |
| Other chronic sinusitis | J32.8 |  |  |  |  |  |  |  |  |  |  |  | 1 | 1 | 1 | 1 |
| Chronic sinusitis, unspecified | J32.9 | 11 | 19 | 18 | 16 | 15 | 15 | **44** | **41** | 42 | 33 | 32 | 32 | 29 |  |  |
| Abscess, furuncle and carbuncle of nose | **J34.0** |  | **1** | **1** | **122** | **127** | **59** | **59** | **43** | **24** | **51** | **47** | **47** | **41** | **25** | **16** |
| Deviated nasal septum | **J34.2** |  |  |  | **5** | **56** | **56** | **56** | **120** | **70** | **51** | **65** | **22** | **26** | **27** | **6** |
| Peritonsillar abscess | J36 |  |  |  |  |  |  |  | 7 | 7 | 7 | 7 | 7 | 7 |  |  |
| Chronic laryngitis | J37.0 | 6 | 6 | 6 |  | 7 | 6 | 6 | 6 | 5 | 7 | 12 | 15 | 11 | 8 | 3 |
| Paralysis of vocal cords and larynx | **J38.0** | **1** | **1** | **1** |  | **16** | **16** | **14** | **14** | **14** | **14** | **14** | **14** | **10** | **10** |  |
| Other diseases of vocal cords | J38.3 |  |  |  |  |  |  |  | 9 | 9 |  |  |  |  |  |  |
| Oedema of larynx | J38.4 |  |  |  |  |  |  | 1 | 1 | 1 | 1 | 1 |  |  |  |  |
| Retropharyngeal and parapharyngeal abscess | J39.0 |  |  |  |  |  |  |  | 16 | 16 | 16 |  |  |  |  |  |
| Bronchitis, not specified as acute or chronic | J40 | 4 | 4 | 4 | 4 | 4 | 4 | 4 | 4 | 1 | 1 |  |  |  |  |  |
| Simple chronic bronchitis | J41.0 |  |  |  |  |  |  |  |  | 2 | 3 | 5 | 5 | 2 | 1 | 1 |
| Unspecified chronic bronchitis | J42 | 11 | 58 | 28 | 41 | 41 | 41 | 30 | 13 | 13 |  |  |  |  |  |  |
| Emphysema, unspecified | J43.9 | 1 | 1 | 1 | 1 |  |  |  |  |  |  |  |  |  |  |  |
| Other chronic obstructive pulmonary disease | **J44** |  |  |  | **65** | **64** | **64** | **64** |  |  |  |  |  |  |  |  |
| Chronic obstructive pulmonary disease with acute lower respiratory infection | J44.0 |  |  |  |  |  |  |  |  |  | 2 | 2 |  | 2 | 1 |  |
| Chronic obstructive pulmonary disease with acute exacerbation, unspecified | J44.1 |  |  |  |  |  |  |  |  |  | 1 | 1 | 1 | 1 | 1 | 1 |
| Other specified chronic obstructive pulmonary disease | J44.8 |  |  |  | 1 |  |  |  |  |  |  |  |  |  |  |  |
| Chronic obstructive pulmonary disease, unspecified | **J44.9** | **35** | **29** | **144** | **242** | **356** | **404** | **382** | **1257** | **1447** | **948** | **631** | **473** | **375** | **212** |  |
| Chronic obstructive pulmonary disease, unspecified, mild | J44.90 |  |  |  |  |  |  |  |  |  |  |  |  | 1 | 1 |  |
| Chronic obstructive pulmonary disease, unspecified, moderate | J44.91 |  |  |  |  |  |  |  |  |  |  |  |  | 10 | 6 | 5 |
| Chronic obstructive pulmonary disease, unspecified, unspecified | J44.99 |  |  |  |  |  |  |  |  |  |  |  |  | 1 |  |  |
| Asthma | J45 | 6 | 5 | 5 | 5 | 5 | 5 | 5 | 4 | 2 | 3 | 2 | 1 | 1 | 1 |  |
| Predominantly allergic asthma | J45.0 |  |  | 40 | 40 |  |  |  |  |  |  |  |  |  |  |  |
| Nonallergic asthma | J45.1 |  |  | 36 |  |  |  |  |  |  |  |  |  |  |  |  |
| Asthma, unspecified | J45.9 |  |  |  |  |  |  |  |  | 5 | 6 | 7 | 6 | 5 | 3 | 2 |
| Status asthmaticus | **J46** |  |  |  | **4** | **4** | **4** |  |  |  |  |  |  |  |  |  |
| Bronchiectasis | J47 | 2 | 2 | 2 | 2 | 2 | 2 | 2 | 2 | 3 | 3 | 2 | 2 | 2 |  |  |
| Pneumonitis due to food and vomit | J69.0 |  | 5 | 5 | 6 | 6 | 1 | **9** | **61** | **89** | 34 | 39 | 36 | 35 | 34 | 1 |
| Pulmonary oedema | J81 |  |  |  |  |  |  |  |  |  |  | 3 | 2 | 2 | 2 | 1 |
| Abscess of lung with pneumonia | J85.1 |  | 5 | 5 | 5 |  |  |  |  |  |  |  |  |  |  |  |
| Acute respiratory failure | J96.0 |  |  |  | 20 |  |  |  |  |  |  |  |  |  |  |  |
| Pulmonary collapse | J98.1 |  |  |  |  |  |  |  |  |  |  | 1 |  |  |  |  |
| Other specified respiratory disorders | J98.8 |  |  |  |  |  |  |  |  |  |  |  |  | 1 |  |  |
| Respiratory disorder, unspecified | J98.9 |  |  |  |  |  |  |  |  |  |  |  |  | 5 | 2 | 1 |

5.1. T1:T2:T3:T4 Renormalization study for comparative analysis

### Table S5-2. The renormalization of the T1:T2:T3:T4 study at Sorok Island.

| year | (T1) | (T2) | (T3) | (T4) | Sum | Mean | | SD | | 95% CI | | [CI | | CI] | | chi-square | | p value | |  |
| --- | --- | --- | --- | --- | --- | --- | --- | --- | --- | --- | --- | --- | --- | --- | --- | --- | --- | --- | --- | --- |
| 2005 | 148 | 95 | 233 | 268 | 744 | 186 | | 78.86 | | 11.41 | | 174.59 | | 264.86 | | 13.5772 | | 0.000229 | |  |
| 2006 | 166 | 111 | 237 | 208 | 722 | 180.5 | | 54.74 | | 8.05 | | 172.45 | | 235.24 | | 3.0793 | | 0.079295 | |  |
| 2007 | 170 | 115 | 252 | 185 | 722 | 180.5 | | 56.37 | | 8.29 | | 172.21 | | 236.87 | | 0.2794 | | 0.597118 | |  |
| 2008 | 207 | 128 | 219 | 148 | 702 | 175.5 | | 44.34 | | 6.61 | | 168.89 | | 219.84 | | 0.3293 | | 0.566073 | |  |
| 2009 | 222 | 135 | 196 | 115 | 668 | 167 | | 50.31 | | 7.69 | | 159.31 | | 217.31 | | 0.0498 | | 0.823418 | |  |
| 2010 | 202 | 170 | 186 | 105 | 663 | 165.75 | | 42.55 | | 6.54 | | 159.21 | | 208.30 | | 6.2203 | | 0.012629 | |  |
| 2011 | 205 | 164 | 170 | 117 | 656 | 164 | | 36.18 | | 5.58 | | 158.42 | | 200.18 | | 0.8918 | | 0.344987 | |  |
| 2012 | 237 | 211 | 103 | 102 | 653 | 163.25 | | 70.95 | | 10.97 | | 152.28 | | 234.20 | | 0.3981 | | 0.528071 | |  |
| 2013 | 269 | 349 | 8 | 23 | 649 | 162.25 | | 172.68 | | 26.79 | | 135.46 | | 334.93 | | 3.7892 | | 0.051583 | |  |
| 2014 | 236 | 349 | 6 | 32 | 623 | 155.75 | | 164.85 | | 26.16 | | 129.59 | | 320.60 | | 9.0547 | | 0.00262 | |  |
| 2015 | 227 | 325 | 7 | 44 | 603 | 150.75 | | 150.82 | | 24.33 | | 126.42 | | 301.57 | | 14.7575 | | 0.000122 | |  |
| 2016 | 207 | 319 | 4 | 61 | 591 | 147.75 | | 142.63 | | 23.25 | | 124.50 | | 290.38 | | 27.7773 | | < 0.00001 | |  |
| 2017 | 193 | 301 | 14 | 55 | 563 | 140.75 | | 131.44 | | 21.96 | | 118.79 | | 272.19 | | 9.1835 | | 0.002442 | |  |
| 2018 | 178 | 303 | 15 | 60 | 556 | 139 | | 129.14 | | 21.66 | | 117.34 | | 268.14 | | 8.2801 | | 0.004008 | |  |
| 2019 | 155 | 297 | 13 | 69 | 534 | 133.5 | | 123.66 | | 21.21 | | 112.29 | | 257.16 | | 10.9433 | | 0.000939 | |  |
|  | **The chi-square is 281.826.** | | | | | |  | |  | |  | |  | |  | |  | |  | |
|  | **The p value is < 0.00001. I t is significant at p < .05.** | | | | | | | | | | | | | | | | | | | |
|  | **The blue means we are dealing with dependent variables; the red, independent.** | | | | | | | | | | | | | | | | | | | |

*Four groups were classified: T1 group is DDS-prescribed (+) with VRD-diagnosed (+) subjects, T2 group is DDS-unprescribed (-) with VRD-diagnosed (+) subjects, T3 group is DDS-prescribed (+) with VRD-undiagnosed (-) subjects, and T4 group is DDS-unprescribed (-) with VRD-undiagnosed (-) subjects. VRD: Viral Respiratory Disease

### Table S5-3. T1:T2:T3:T4 study for 2005, 2010, 2014-2019 Dealing Dependent variables at Sorok Island

| year | (T1) | (T2) | (T3) | (T4) | Sum | Mean | SD | 95% CI | [CI | CI] | chi-square | p value |
| --- | --- | --- | --- | --- | --- | --- | --- | --- | --- | --- | --- | --- |
| 2005 | 148 | 95 | 233 | 268 | 744 | 186 | 78.86 | 11.41 | 174.59 | 264.86 | 13.5772 | 0.000229 |
| 2010 | 202 | 170 | 186 | 105 | 663 | 165.75 | 42.55 | 6.54 | 159.21 | 208.30 | 6.2203 | 0.012629 |
| 2014 | 236 | 349 | 6 | 32 | 623 | 155.75 | 164.85 | 26.16 | 129.59 | 320.60 | 9.0547 | 0.00262 |
| 2015 | 227 | 325 | 7 | 44 | 603 | 150.75 | 150.82 | 24.33 | 126.42 | 301.57 | 14.7575 | 0.000122 |
| 2016 | 207 | 319 | 4 | 61 | 591 | 147.75 | 142.63 | 23.25 | 124.50 | 290.38 | 27.7773 | < 0.00001 |
| 2017 | 193 | 301 | 14 | 55 | 563 | 140.75 | 131.44 | 21.96 | 118.79 | 272.19 | 9.1835 | 0.002442 |
| 2018 | 178 | 303 | 15 | 60 | 556 | 139 | 129.14 | 21.66 | 117.34 | 268.14 | 8.2801 | 0.004008 |
| 2019 | 155 | 297 | 13 | 69 | 534 | 133.5 | 123.66 | 21.21 | 112.29 | 257.16 | 10.9433 | 0.000939 |

### Table S5-4. One-Way ANOVA Calculator for Independent Measures of T1:T2:T3:T4 study for 2005, 2010, 2014-2019 at Sorok Island

| \|  \| T1 \| T2 \| T3 \| T4 \| Total \| \| --- \| --- \| --- \| --- \| --- \| --- \| \| N \| 8 \| 8 \| 8 \| 8 \| 32 \| \| ∑X \| 1546 \| 2159 \| 478 \| 694 \| 4877 \| \| Mean \| 193.25 \| 269.875 \| 59.75 \| 86.75 \| 152.406 \| \| ∑X^2^ \| 305740 \| 637731 \| 89576 \| 100916 \| 1133963 \| \| Std.Dev. \| 31.5674 \| 88.6976 \| 93.3622 \| 76.2622 \| 112.2609 \|  \| ***Source*** \| ***SS*** \| ***df*** \| ***MS*** \|  \| \| --- \| --- \| --- \| --- \| --- \| \| Between-treatments \| 226904.3438 \| 3 \| 75634.7813 \| *F* = 12.93112 \| \| Within-treatments \| 163773.375 \| 28 \| 5849.0491 \|  \| \| Total \| 390677.7188 \| 31 \|  \|  \|   The *f*-ratio value is 12.93112. The *p* value is .000017. The result is significant at *p* < .05. |
| --- | --- | --- | --- | --- | --- | --- | --- | --- | --- | --- | --- | --- | --- | --- | --- | --- | --- | --- | --- | --- | --- | --- | --- | --- | --- | --- | --- | --- | --- | --- | --- | --- | --- | --- | --- | --- | --- | --- | --- | --- | --- | --- | --- | --- | --- | --- | --- | --- | --- | --- | --- | --- | --- | --- | --- | --- |

### Table S5-5. Post hoc Tukey HSD

| *Pairwise Comparisons* | | HSD_.05_ = 104.4047 HSD_.01_ = 130.5897 | Q_.05_ = 3.8612  Q_.01_ = 4.8296 |
| --- | --- | --- | --- |
| T_1_:T_2_ | M_1_ = 193.25 M_2_ = 269.88 | 76.63 | Q = 2.83 (*p* = .21070) |
| T_1_:T_3_ | M_1_ = 193.25 M_3_ = 59.75 | 133.50 | Q = 4.94 (*p* = .00827) |
| T_1_:T_4_ | M_1_ = 193.25 M_4_ = 86.75 | 106.50 | Q = 3.94 (*p* = .04429) |
| T_2_:T_3_ | M_2_ = 269.88 M_3_ = 59.75 | 210.13 | Q = 7.77 (*p* = .00004) |
| T_2_:T_4_ | M_2_ = 269.88 M_4_ = 86.75 | 183.13 | Q = 6.77 (*p* = .00027) |
| T_3_:T_4_ | M_3_ = 59.75 M_4_ = 86.75 | 27.00 | Q = 1.00 (*p* = .89383) |

We excluded the T1:T2 and T3:T4 tests.

### Table S5-6. One-Way ANOVA Calculator for repeated Measures of T1:T2:T3:T4 study for 2005, 2010, 2014-2019 Dealing Dependent variables at Sorok Island

| \|  \| T1 \| T2 \| T3 \| T4 \| Total \| \| --- \| --- \| --- \| --- \| --- \| --- \| \| N \| 8 \| 8 \| 8 \| 8 \| 32 \| 32 \| \| ∑X \| 1546 \| 2159 \| 478 \| 694 \| 4877 \| 4877 \| \| Mean \| 193.25 \| 269.875 \| 59.75 \| 86.75 \| 152.406 \| 152.406 \| \| ∑X^2^ \| 305740 \| 637731 \| 89576 \| 100916 \| 1133963 \| 1133963 \| \| Std.Dev. \| 31.5674 \| 88.6976 \| 93.3622 \| 76.2622 \| 112.2609 \| 112.2609 \|  \| *Source* \| *SS* \| *df* \| *MS* \|  \| \| --- \| --- \| --- \| --- \| --- \| \| Between-treatments \| 226904.3438 \| 3 \| 75634.7813 \| *F* = 10.20041 \| \| Within-treatments \| 163773.375 \| 28 \| 5849.0491 \|  \| \| Total \| 155712.4063 \| 21 \| 7414.8765 \|  \|   The *F*-ratio value is 10.20041. The *p* value is .000238. The result is significant at *p* < .05. |
| --- | --- | --- | --- | --- | --- | --- | --- | --- | --- | --- | --- | --- | --- | --- | --- | --- | --- | --- | --- | --- | --- | --- | --- | --- | --- | --- | --- | --- | --- | --- | --- | --- | --- | --- | --- | --- | --- | --- | --- | --- | --- | --- | --- | --- | --- | --- | --- | --- | --- | --- | --- | --- | --- | --- | --- | --- | --- | --- | --- | --- | --- |

Fig. S7. Dependent Study Result of T1 T2 T3 T4


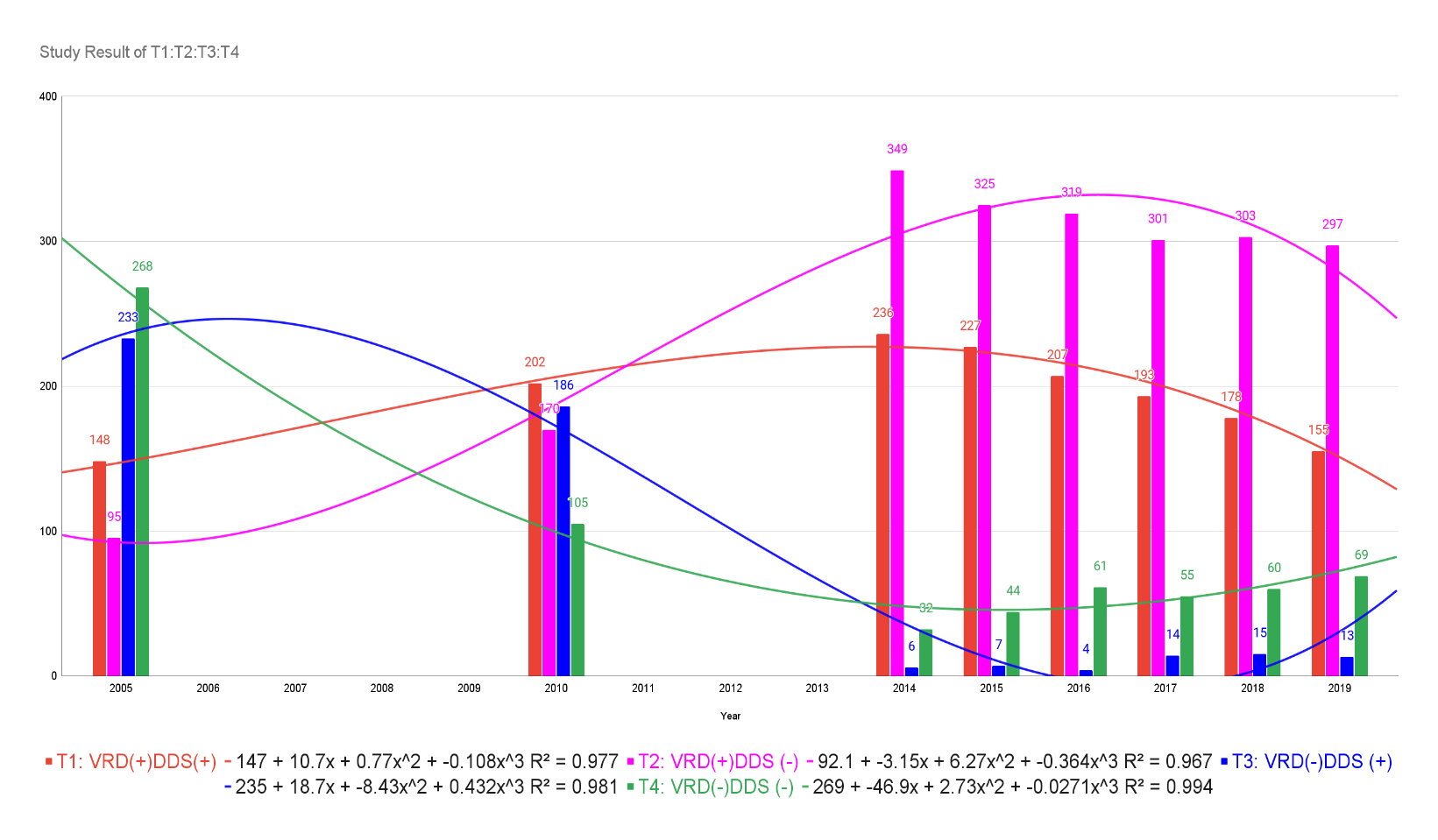


5.2. Addressing dependent variables for the T1:T3 study at Sorok Island

Table S5-7. T test for T1:T3

| Independent Means | Dependent Means |
| --- | --- |
| Significance Level: 0.05, Two-tailed hypothesis. | Significance Level: 0.05, Two-tailed hypothesis. |
| The t value is 3.83133. The p value is .001834. The result is significant at p < .05. | The value of *t* is -3.402961. The value of *p* is .0114. The result is significant at p < .05. |
| Difference Scores Calculations  Treatment 1  N1: 8  df1 = N - 1 = 8 - 1 = 7  M1: 193.25  SS1: 6975.5  s21 = SS1/(N - 1) = 6975.5/(8-1) = 996.5  Treatment 2  N2: 8  df2 = N - 1 = 8 - 1 = 7  M2: 59.75  SS2: 61015.5  s22 = SS2/(N - 1) = 61015.5/(8-1) = 8716.5  T value Calculation  s2p = ((df1/(df1 + df2)) * s21) + ((df2/(df2 + df2)) * s22) = ((7/14) * 996.5) + ((7/14) * 8716.5) = 4856.5  s2M1 = s2p/N1 = 4856.5/8 = 607.06  s2M2 = s2p/N2 = 4856.5/8 = 607.06  t = (M1 - M2)/√(s2M1 + s2M2) = 133.5/√1214.12 = 3.83 | Difference Scores Calculations  *Mean*: -133.5 *μ* = 0 *S^2^* = *SS⁄df* = 86186/(8-1) = 12312.29 *S^2^_M_* = *S^2^/N* = 12312.29/8 = 1539.04 *S_M_* = √*S^2^_M_* = √1539.04 = 39.23  T value Calculation  *t* = (*M* - *μ*)/*S_M_* = (-133.5 - 0)/39.23 = -3.4 |

Table S5-8. Effect Size Calculator for T Test for T1:T3

| T1  Mean (M):193.25  Standard deviation (s):6975.5  Sample size (n):8 | T3  Mean (M):59.75  Standard deviation (s):61015.5  Sample size (n):8 |
| --- | --- |
| Cohen's d = (59.75 - 193.25) ⁄ 43425.50426 = 0.003074.  Glass's delta = (59.75 - 193.25) ⁄ 6975.5 = 0.019138.  Hedges' g = (59.75 - 193.25) ⁄ 43425.50426 = 0.003074. | |

Fig. S8. Dependent Study Result of T1 T3


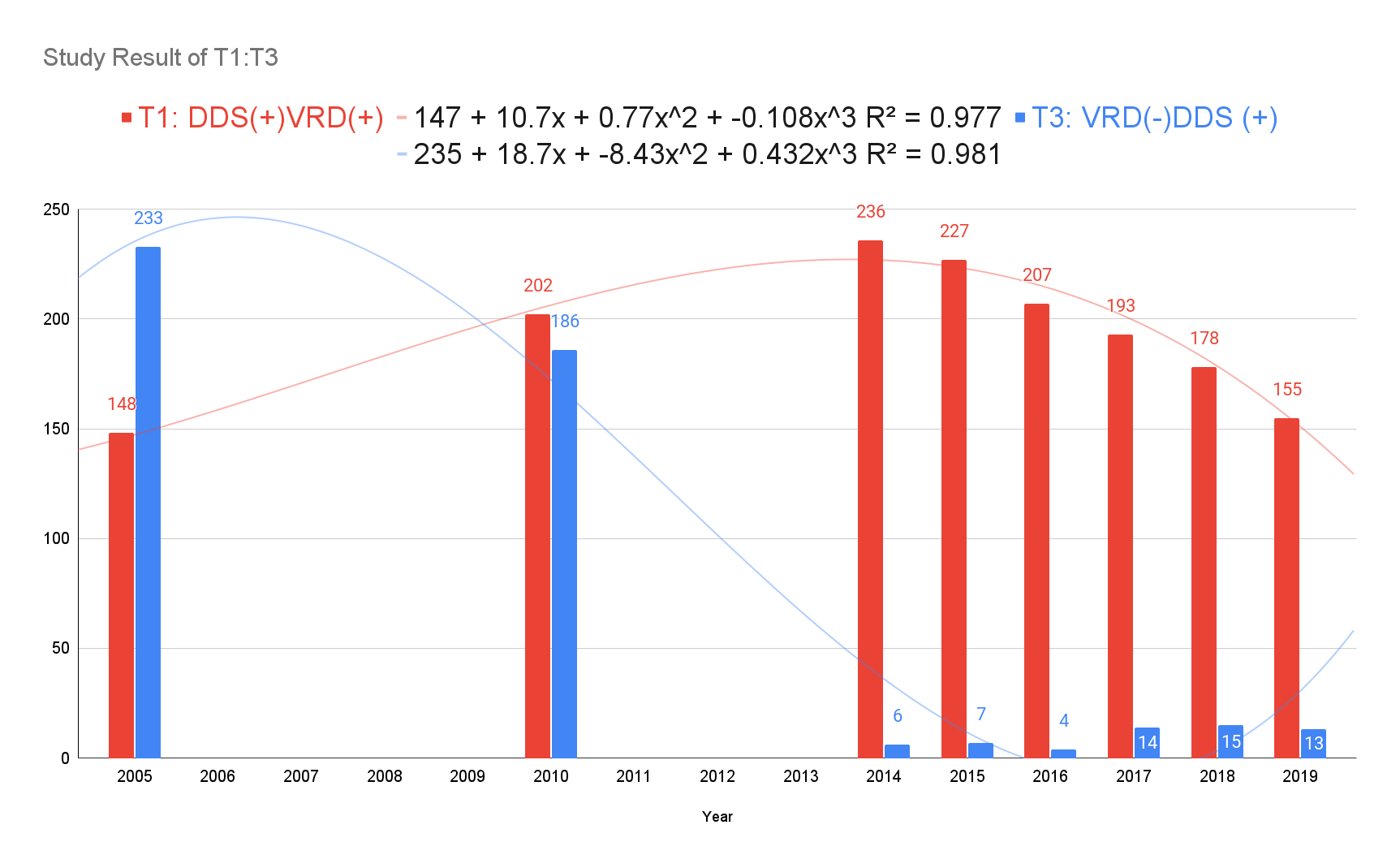


Table S5-8. T test for T1:T4

| Independent Means | Dependent Means |
| --- | --- |
| Significance Level: 0.05, Two-tailed hypothesis. | Significance Level: 0.05, Two-tailed hypothesis. |
| The t value is 3.64959. The p value is .002627. The result is significant at p < .05. | The value of t is -3.015378. The value of p is .01951. The result is significant at p < .05. |
| Difference Scores Calculations  Treatment 1  N1: 8  df1 = N - 1 = 8 - 1 = 7  M1: 193.25  SS1: 6975.5  s21 = SS1/(N - 1) = 6975.5/(8-1) = 996.5  Treatment 2  N2: 8  df2 = N - 1 = 8 - 1 = 7  M2: 86.75  SS2: 40711.5  s22 = SS2/(N - 1) = 40711.5/(8-1) = 5815.93  T value Calculation  s2p = ((df1/(df1 + df2)) * s21) + ((df2/(df2 + df2)) * s22) = ((7/14) * 996.5) + ((7/14) * 5815.93) = 3406.21  s2M1 = s2p/N1 = 3406.21/8 = 425.78  s2M2 = s2p/N2 = 3406.21/8 = 425.78  t = (M1 - M2)/√(s2M1 + s2M2) = 106.5/√851.55 = 3.65 | Difference Scores Calculations  Mean: -106.5  μ = 0  S2 = SS⁄df = 69856/(8-1) = 9979.43  S2 M = S2/N = 9979.43/8 = 1247.43  SM = √S2 M = √1247.43 = 35.32  T value Calculation  t = (M - μ)/SM = (-106.5 - 0)/35.32 = -3.02 |

Table S5-9. Effect Size Calculator for T Test for T1:T4

| T1  Mean (M):193.25  Standard deviation (s):6975.5  Sample size (n):8 | T3  Mean (M):86.75  Standard deviation (s):40711.5  Sample size (n):8 |
| --- | --- |
| Cohen's d = (86.75 - 193.25) ⁄ 29206.881317 = 0.003646.  Glass's delta = (86.75 - 193.25) ⁄ 6975.5 = 0.015268.  Hedges' g = (86.75 - 193.25) ⁄ 29206.881317 = 0.003646. | |

Fig. S9. Dependent Study Result of T1:T4


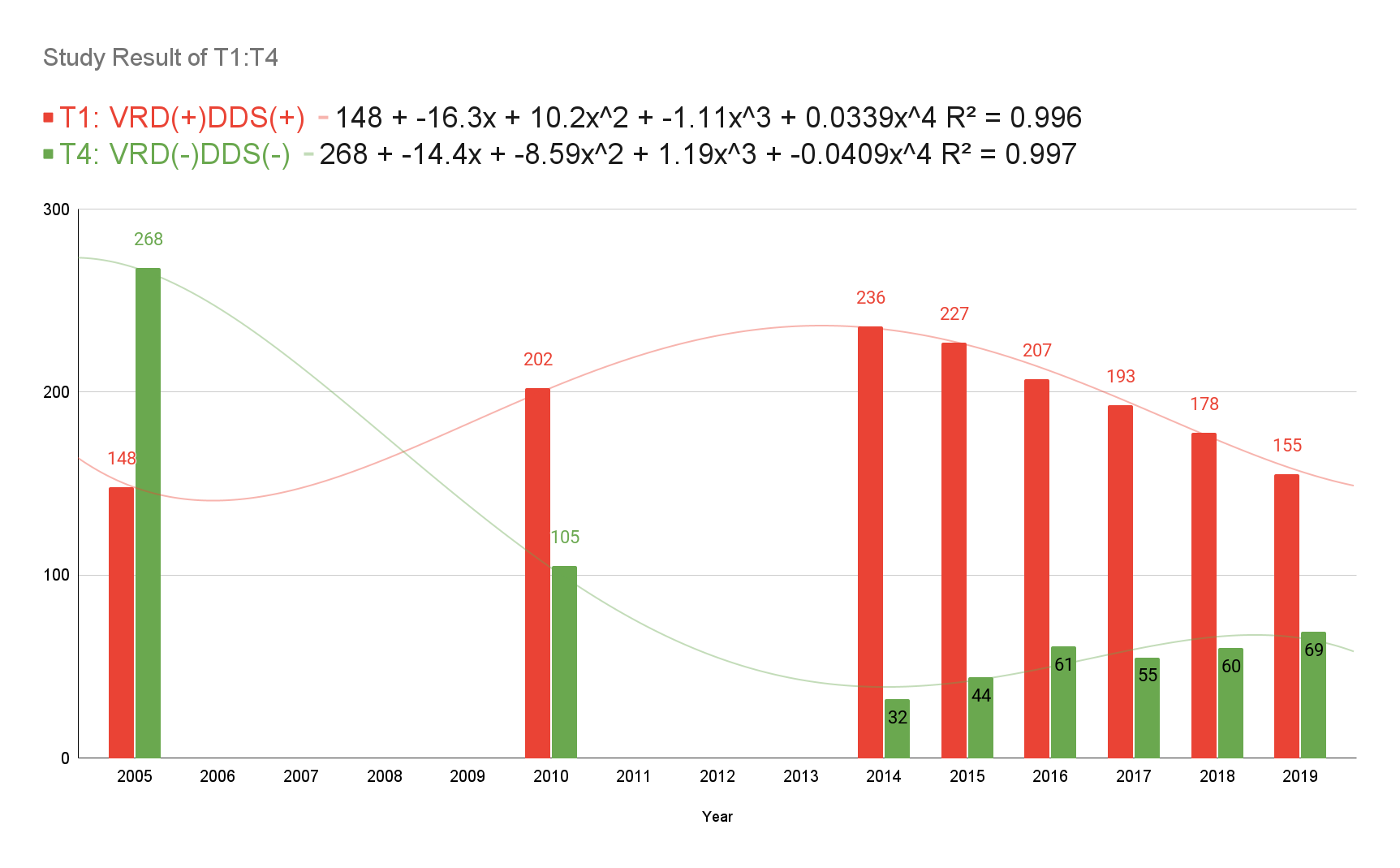


Table S5-10. T test for T2:T3

| Independent Means | Dependent Means |
| --- | --- |
| Significance Level: 0.05, Two-tailed hypothesis. | Significance Level: 0.05, Two-tailed hypothesis. |
| The t value is 4.6151. The p value is .000401. The result is significant at p < .05. | The value of *t* is -3.402961. The value of *p* is .0114. The result is significant at p < .05. |
| Difference Scores Calculations  Treatment 1  N1: 8  df1 = N - 1 = 8 - 1 = 7  M1: 269.88  SS1: 55070.88  s21 = SS1/(N - 1) = 55070.88/(8-1) = 7867.27  Treatment 2  N2: 8  df2 = N - 1 = 8 - 1 = 7  M2: 59.75  SS2: 61015.5  s22 = SS2/(N - 1) = 61015.5/(8-1) = 8716.5  T value Calculation  s2p = ((df1/(df1 + df2)) * s21) + ((df2/(df2 + df2)) * s22) = ((7/14) * 7867.27) + ((7/14) * 8716.5) = 8291.88  s2M1 = s2p/N1 = 8291.88/8 = 1036.49  s2M2 = s2p/N2 = 8291.88/8 = 1036.49  t = (M1 - M2)/√(s2M1 + s2M2) = 210.12/√2072.97 = 4.62 | Difference Scores Calculations  *Mean*: -133.5 *μ* = 0 *S^2^* = *SS⁄df* = 86186/(8-1) = 12312.29 *S^2^_M_* = *S^2^/N* = 12312.29/8 = 1539.04 *S_M_* = √*S^2^_M_* = √1539.04 = 39.23  T value Calculation  *t* = (*M* - *μ*)/*S_M_* = (-133.5 - 0)/39.23 = -3.4 |

Table S5-11. Effect Size Calculator for T Test for T2:T3

| T2  Mean (M):269.88  Standard deviation (s):55070.88  Sample size (n):8 | T3  Mean (M):59.75  Standard deviation (s):61015.5  Sample size (n):8 |
| --- | --- |
| Cohen's d = (59.75 - 269.88) ⁄ 58119.244077 = 0.003615.  Glass's delta = (59.75 - 269.88) ⁄ 55070.88 = 0.003816.  Hedges' g = (59.75 - 269.88) ⁄ 58119.244077 = 0.003615. | |

Fig. S10. Dependent Study Result of T2 T3


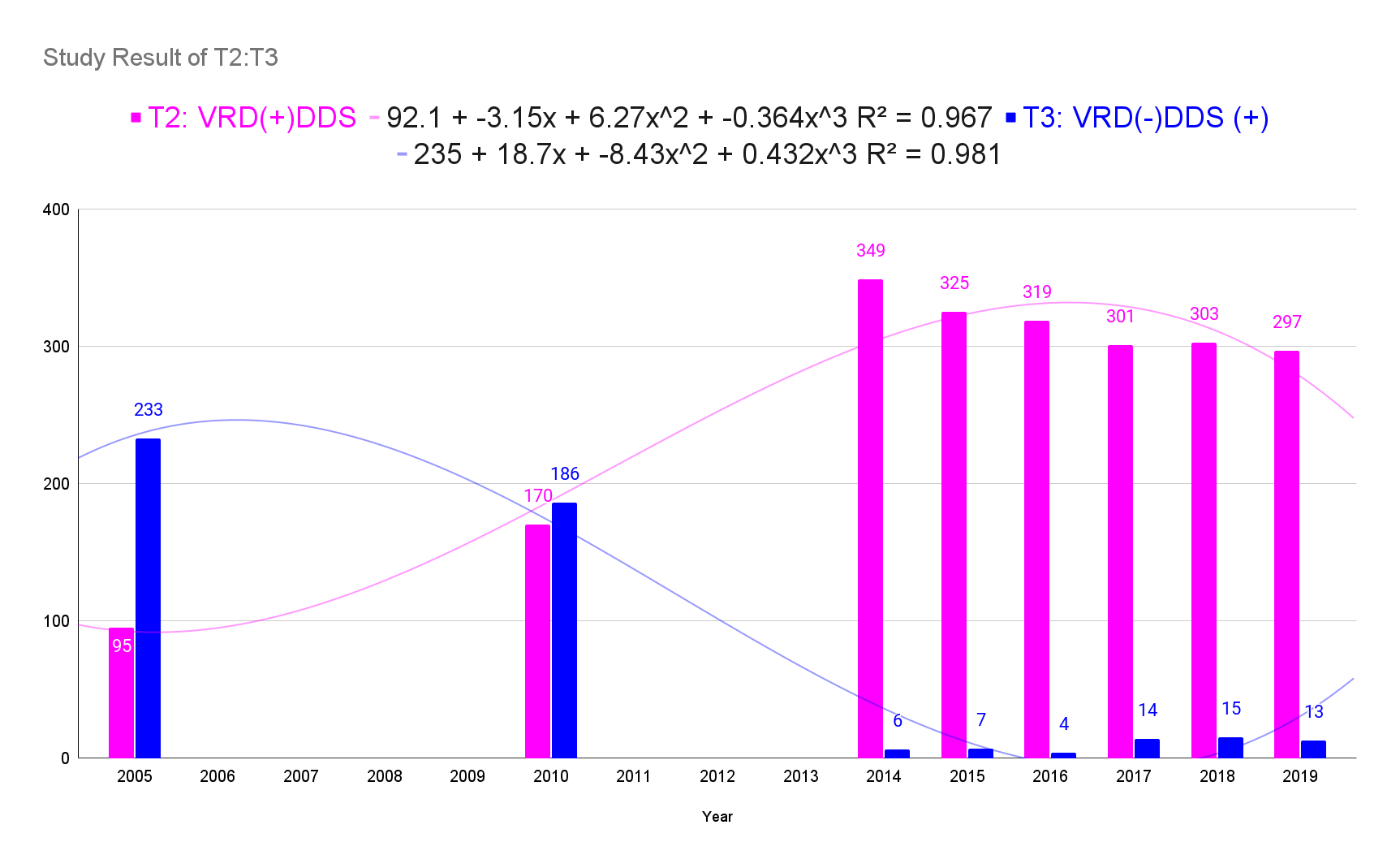


Table S5-12. T test for T2:T4

| Independent Means | Dependent Means |
| --- | --- |
| Significance Level: 0.05, Two-tailed hypothesis. | Significance Level: 0.05, Two-tailed hypothesis. |
| The t value is 4.42791. The p value is .000573. The result is significant at p < .05. | The value of *t* is -3.402961. The value of *p* is .0114. The result is significant at p < .05. |
| Difference Scores Calculations  Treatment 1  N1: 8  df1 = N - 1 = 8 - 1 = 7  M1: 269.88  SS1: 55070.88  s21 = SS1/(N - 1) = 55070.88/(8-1) = 7867.27  Treatment 2  N2: 8  df2 = N - 1 = 8 - 1 = 7  M2: 86.75  SS2: 40711.5  s22 = SS2/(N - 1) = 40711.5/(8-1) = 5815.93  T value Calculation  s2p = ((df1/(df1 + df2)) * s21) + ((df2/(df2 + df2)) * s22) = ((7/14) * 7867.27) + ((7/14) * 5815.93) = 6841.6  s2M1 = s2p/N1 = 6841.6/8 = 855.2  s2M2 = s2p/N2 = 6841.6/8 = 855.2  t = (M1 - M2)/√(s2M1 + s2M2) = 183.12/√1710.4 = 4.43 | Difference Scores Calculations  *Mean*: -133.5 *μ* = 0 *S^2^* = *SS⁄df* = 86186/(8-1) = 12312.29 *S^2^_M_* = *S^2^/N* = 12312.29/8 = 1539.04 *S_M_* = √*S^2^_M_* = √1539.04 = 39.23  T value Calculation  *t* = (*M* - *μ*)/*S_M_* = (-133.5 - 0)/39.23 = -3.4 |

Table S5-13. Effect Size Calculator for T Test for T2:T4

| T2  Mean (M):269.88  Standard deviation (s):55070.88  Sample size (n):8 | T4  Mean (M):86.75  Standard deviation (s):40711.5  Sample size (n):8 |
| --- | --- |
| Cohen's d = (86.75 - 269.88) ⁄ 48426.3774 = 0.003782.  Glass's delta = (86.75 - 269.88) ⁄ 55070.88 = 0.003325.  Hedges' g = (86.75 - 269.88) ⁄ 48426.3774 = 0.003782. | |

Fig. S11. Dependent Study Result of T2 T4


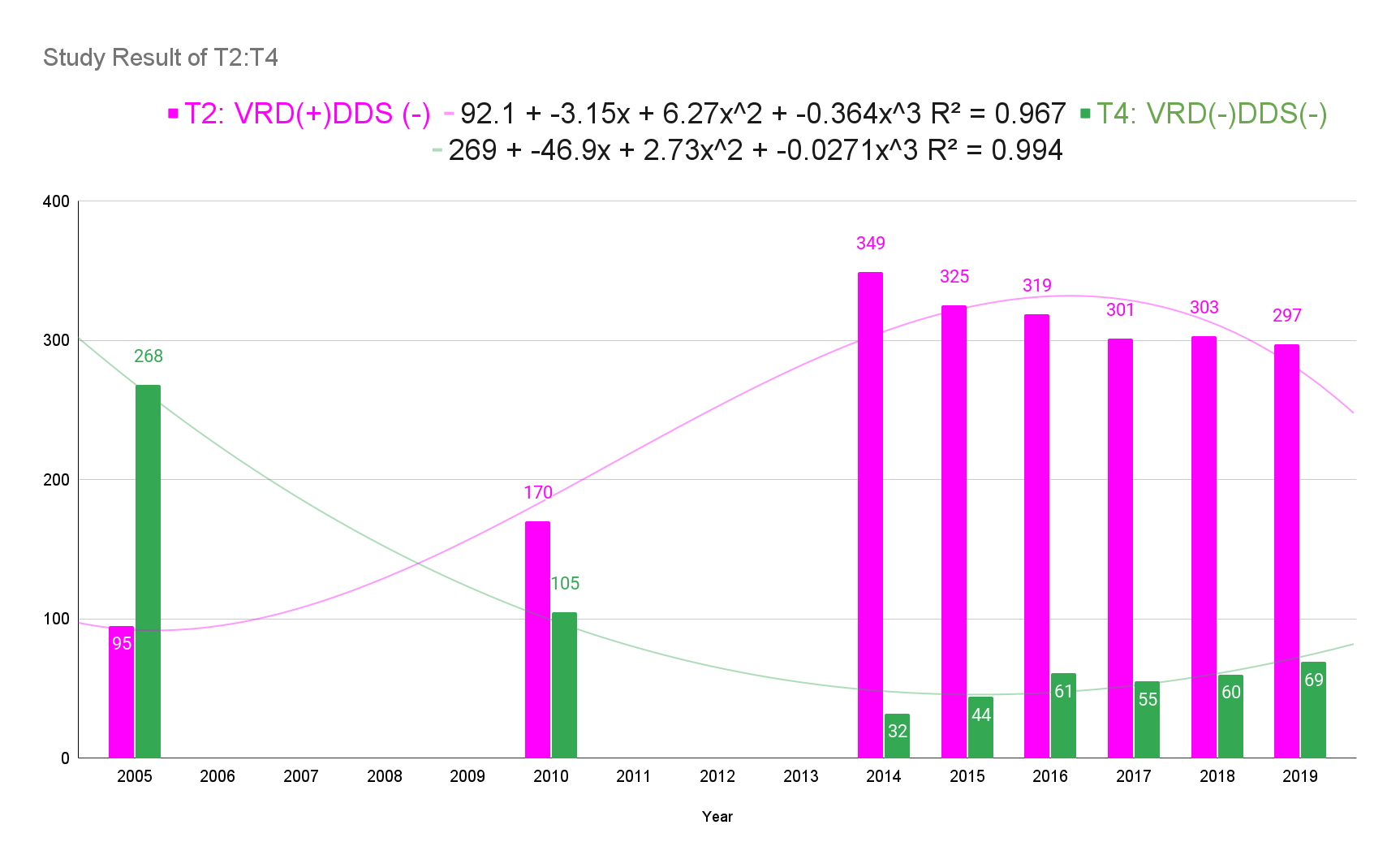


5.3. The average age of death on Sorok Island.

### Fig. S12. The average age of death on Sorok Island.

**
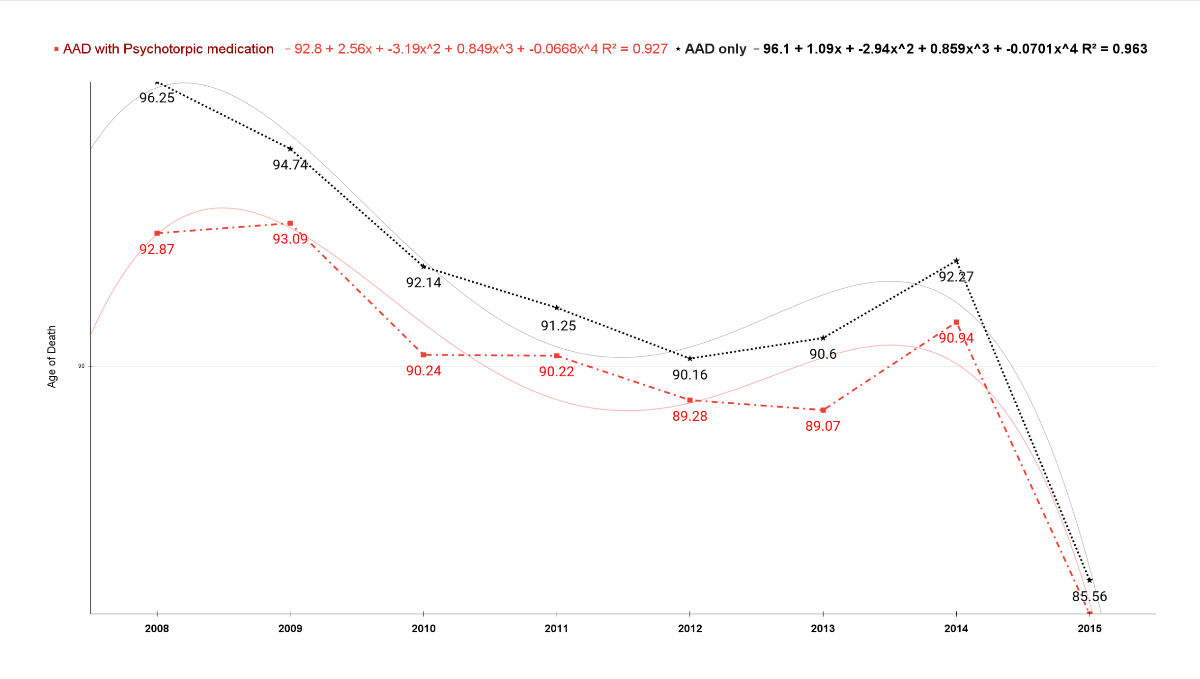
**

The expectancy of Hansen’s disease (HD) patients with Alzheimer’s disease (AD) taking AChEIs or memantine with psychotropic medicines at Sorok Island. The mean ages of death decrease. The mean ages of deaths without taking additional psychotropic drugs^*^ are black. The life expectancy trends of HD patients taking other psychotropic medications (red) decreased. The life expectancy trends of HD patients taking anti-Alzheimer’s disease drugs^**^ (AAD) were decreased on Sorok Island (black), and those taking AAD with psychotropic medicines (red) were reduced more.

^*^ For psychologic symptoms of Alzheimer’s disease: Haloperidol- 167903ATB 167904ATB 167905ATB 167906ATB 167908ATB 167908ATB 168030BIJ, Risperidone- 224201ATB 224201ATD 224202ATB 224202ATD 224203ATB 224204ATB 224205BIJ 224206BIJ, Quetiapine- 378601ATB 378602ATB 378603ATB 378604ATB 378605ATB 378605ATR 378606ATR 378607ATR 378608ATR 378608ATR 378610ATB, Olanzapine- 204001ATB 204001ATD 204002ATB 204002ATD 204004ATB 204005ATB, Aripiprazole- 451501ATB 451501ATD 451502ATB 451502ATD 451503ATB 451504ATB 451505ATB 451506BIJ 451507BIJ, Oxcarbazepine- 206330ASS 206301ATB 206302ATB 206303ATB, Fluvoxamine- 162501ATB 162502ATB, Escitalopram- 474801ATB 474802ATB 474803ATB 474804ATB, Trazodone- 242901ACH 242901ATB 242902ATB 242903ATR, Sertraline- 227001ATB 227002ATB 227003ATB, Escitalopram- 474801ATB 474802ATB 474803ATB 474804ATB, Fluoxetine- 161501ACH 161501ATB 161502ACH 161502ATB 161502ATD

^**^ For symptomatic relief of Alzheimer’s disease: Donepezil hydrochloride- 148603ATB 148602ATD 148602ATB 148601ATD 148601ATB 643401ATD 643402ATD, rivastigmine- 224501ACH 224503ACH 224504ACH 224505ACH 224506CPC 224507CPC 224508CPC, galantamine- 385203ACR 385203ATR 385204ACR 385204ATR 385205ACR 385205ATR, N-methyl-D-aspartate (NMDA) receptor antagonist- 190031ALQ 190001ATB 190003ATD 190004ATB 190004ATD.

**Section 6. Statistical Analysis for the Correlation OR Coefficient of determination**

6.1 Bronchitis and Factor

**Pearson** **Correlation Coefficient Calculator**

The value of R is -0.8966.

| X Values  37  84  34  47  47  47  36  1047  1364  1311  708  450  444  438  8 | Y Values  271  265  266  252  234  188  157  103  23  18  75  101  125  160  220 | 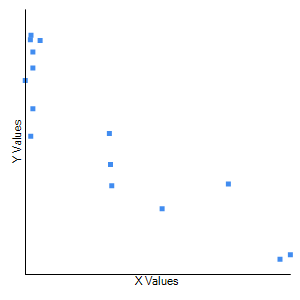 |
| --- | --- | --- |
|  |  |  |

| X - M_x_ | Y - M_y_ | (X - M_x_)^2^ | (Y - M_y_)^2^ | (X - M_x_)(Y - M_y_) |
| --- | --- | --- | --- | --- |
| -369.800  -322.800  -372.800  -359.800  -359.800  -359.800  -370.800  640.200  957.200  904.200  301.200  43.200  37.200  31.200  -398.800  Mx: 406.800 | 107.133  101.133  102.133  88.133  70.133  24.133  -6.867  -60.867  -140.867  -145.867  -88.867  -62.867  -38.867  -3.867  56.133  My: 163.867 | 136752.040  104199.840  138979.840  129456.040  129456.040  129456.040  137492.640  409856.040  916231.840  817577.640  90721.440  1866.240  1383.840  973.440  159041.440  Sum: 3303444.400 | 11477.551  10227.951  10431.218  7767.484  4918.684  582.418  47.151  3704.751  19843.418  21277.084  7897.284  3952.218  1510.618  14.951  3150.951  Sum: 106803.733 | -39617.907  -32645.840  -38075.307  -31710.373  -25233.973  -8683.173  2546.160  -38966.840  -134837.573  -131892.640  -26766.640  -2715.840  -1445.840  -120.640  -22385.973  Sum: -532552.400 |

| **Result Details & Calculation**  *X Values* ∑ = 6102 Mean = 406.8 ∑(X - M_x_)^2^ = SS_x_ = 3303444.4  *Y Values* ∑ = 2458 Mean = 163.867 ∑(Y - M_y_)^2^ = SS_y_ = 106803.733  *X and Y Combined* *N* = 15 ∑(X - M_x_)(Y - M_y_) = -532552.4  *R Calculation* r = ∑((X - M_y_)(Y - M_x_))/√((SS_x_)(SS_y_))  r = -532552.4/√((3303444.4)(106803.733)) = -0.8966  *Meta Numerics (cross-check)* r = -0.8966 | Key  X: X Values  Y: Y Values  Mx: Mean of X Values  My: Mean of Y Values  X - Mx & Y - My: Deviation scores  (X - Mx)2 & (Y - My)2: Deviation Squared  (X - Mx)(Y - My): Product of Deviation Scores |
| --- | --- |

The value of R is -0.8966.

This is a strong negative correlation, which means that high X variable scores are associated with low Y variable scores (and vice versa).

The value of R^2^, the coefficient of determination, is 0.8039.

The P value is.000304. The result is significant at p <.05.

**Spearman's Rho Calculator**

The value of *r_s_* is -0.80287.

*r_s_* = -0.80287, *p* (2-tailed) = 0.00031.

By normal standards, the association between the two variables was considered statistically significant.

| X Values | Y Values | *X_Ra_* | *X_Ra_ - M_x_* | *Y_Ra_* | *Y_Ra_ - M_y_* | *Sum Diffs* |
| --- | --- | --- | --- | --- | --- | --- |
| 37  84  34  47  47  47  36  1047  1364  1311  708  450  444  438  8 | 271  265  266  252  234  188  157  103  23  18  75  101  125  160  220 | 4.00  8.00  2.00  6.00  6.00  6.00  3.00  13.00  15.00  14.00  12.00  11.00  10.00  9.00  1.00 | -4.00  0.00  -6.00  -2.00  -2.00  -2.00  -5.00  5.00  7.00  6.00  4.00  3.00  2.00  1.00  -7.00 | 15.00  13.00  14.00  12.00  11.00  9.00  7.00  5.00  2.00  1.00  3.00  4.00  6.00  8.00  10.00 | 7.00  5.00  6.00  4.00  3.00  1.00  -1.00  -3.00  -6.00  -7.00  -5.00  -4.00  -2.00  0.00  2.00 | -28.00  0.00  -36.00  -8.00  -6.00  -2.00  5.00  -15.00  -42.00  -42.00  -20.00  -12.00  -4.00  0.00  -14.00 |

| Calculation  R = CoVariance/(X_Ra_ St. Dev. * Y_Ra_ St. Dev.)  Key  X_Ra_ = Ranks of X Values; Y_Ra_ = Ranks of Y Values X_Ra_ - M_x_ = X rank minus mean of X ranks Y_Ra_ - M_y_ = Y rank minus mean of Y ranks Sum Diffs = (X_Ra_ - M_x_) * (Y_Ra_ - M_y_) | Result Details  *X Ranks* Mean: 8 Standard Dev: 4.46  *Y Ranks* Mean: 8 Standard Dev: 4.47  *Combined* Covariance = -224/14 = -16 R = -16/(4.46 * 4.47) = -0.803 |
| --- | --- |

*r_s_* = -0.80287, *p* (2-tailed) = 0.00031.

By normal standards, the association between the two variables was considered statistically significant.

6.2. Pneumonia and Factors

**Pearson Correlation Coefficient Calculator**

The value of R is -0.4402.

| X Values  41  94  62  366  264  249  419  415  801  266  101  73  39  24  13 | Y Values  271  265  266  252  234  188  157  103  23  18  75  101  125  160  220 | 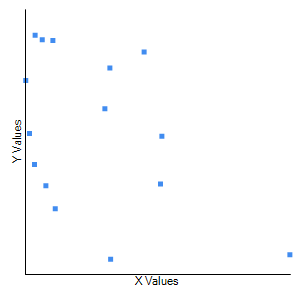 |
| --- | --- | --- |
|  |  |  |

| X - M_x_ | Y - M_y_ | (X - M_x_)^2^ | (Y - M_y_)^2^ | (X - M_x_)(Y - M_y_) |
| --- | --- | --- | --- | --- |
| -174.133  -121.133  -153.133  150.867  48.867  33.867  203.867  199.867  585.867  50.867  -114.133  -142.133  -176.133  -191.133  -202.133  Mx: 215.133 | 107.133  101.133  102.133  88.133  70.133  24.133  -6.867  -60.867  -140.867  -145.867  -88.867  -62.867  -38.867  -3.867  56.133  My: 163.867 | 30322.418  14673.284  23449.818  22760.751  2387.951  1146.951  41561.618  39946.684  343239.751  2587.418  13026.418  20201.884  31022.951  36531.951  40857.884  Sum: 663717.733 | 11477.551  10227.951  10431.218  7767.484  4918.684  582.418  47.151  3704.751  19843.418  21277.084  7897.284  3952.218  1510.618  14.951  3150.951  Sum: 106803.733 | -18655.484  -12250.618  -15640.018  13296.382  3427.182  817.316  -1399.884  -12165.218  -82529.084  -7419.751  10142.649  8935.449  6845.716  739.049  -11346.418  Sum: -117202.733 |

| **Result Details & Calculation** *X Values* ∑ = 3227 Mean = 215.133 ∑(X - M_x_)^2^ = SS_x_ = 663717.733  *Y Values* ∑ = 2458 Mean = 163.867 ∑(Y - M_y_)^2^ = SS_y_ = 106803.733  *X and Y Combined* *N* = 15 ∑(X - M_x_)(Y - M_y_) = -117202.733  *R Calculation* r = ∑((X - M_y_)(Y - M_x_))/√((SS_x_)(SS_y_))  r = -117202.733/√((663717.733)(106803.733)) = -0.4402  *Meta Numerics (cross-check)* r = -0.4402 | Key  *X*: X Values *Y*: Y Values *M_x_*: Mean of X Values *M_y_*: Mean of Y Values *X - M*_x_ & *Y - M_y_*: Deviation scores *(X - M_x_*)^2^ & *(Y - M_y_)^2^*: Deviation Squared *(X - M_x_)(Y - M_y_*): Product of Deviation Scores |
| --- | --- |

The value of R is -0.4402.

Although technically a negative correlation, the relationship between your variables is only weak (*nb.* The nearer the value is to zero, the weaker the relationship).

The value of R^2^, the coefficient of determination, is 0.1938.

The P value is .100742. The result is *not* significant at p <.05.

**Spearman's Rho Calculator**

The value of *r_s_* is -0.38571.

*r_s_* = -0.38571, *p* (2-tailed) = 0.15563.

By normal standards, the association between the two variables would not be considered statistically significant.

| X Values | Y Values | *X_Ra_* | *X_Ra_ - M_x_* | *Y_Ra_* | *Y_Ra_ - M_y_* | *Sum Diffs* |
| --- | --- | --- | --- | --- | --- | --- |
| 41  94  62  366  264  249  419  415  801  266  101  73  39  24  13 | 271  265  266  252  234  188  157  103  23  18  75  101  125  160  220 | 4.00  7.00  5.00  12.00  10.00  9.00  14.00  13.00  15.00  11.00  8.00  6.00  3.00  2.00  1.00 | -4.00  -1.00  -3.00  4.00  2.00  1.00  6.00  5.00  7.00  3.00  0.00  -2.00  -5.00  -6.00  -7.00 | 15.00  13.00  14.00  12.00  11.00  9.00  7.00  5.00  2.00  1.00  3.00  4.00  6.00  8.00  10.00 | 7.00  5.00  6.00  4.00  3.00  1.00  -1.00  -3.00  -6.00  -7.00  -5.00  -4.00  -2.00  0.00  2.00 | -28.00  -5.00  -18.00  16.00  6.00  1.00  -6.00  -15.00  -42.00  -21.00  0.00  8.00  10.00  0.00  -14.00 |

| Calculation  R = CoVariance/(X_Ra_ St. Dev. * Y_Ra_ St. Dev.)  Key  X_Ra_ = Ranks of X Values; Y_Ra_ = Ranks of Y Values X_Ra_ - M_x_ = X rank minus mean of X ranks Y_Ra_ - M_y_ = Y rank minus mean of Y ranks Sum Diffs = (X_Ra_ - M_x_) * (Y_Ra_ - M_y_) | Result Details  *X Ranks Mean: 8 Standard Dev: 4.47  Y Ranks Mean: 8 Standard Dev: 4.47  Combined Covariance = -108/14 = -7.71 R = -7.71/(4.47 * 4.47) = -0.386* |
| --- | --- |

*r_s_* = -0.38571, *p* (2-tailed) = 0.15563.

By normal standards, the association between the two variables was not considered statistically significant.

6.3. COPD and Factor

**Pearson Correlation Coefficient Calculator**

The value of R is -0.8161.

| X Values  37  31  146  309  422  470  448  1259  1450  951  633  475  377  212  0 | Y Values  271  265  266  252  234  188  157  103  23  18  75  101  125  160  220 | 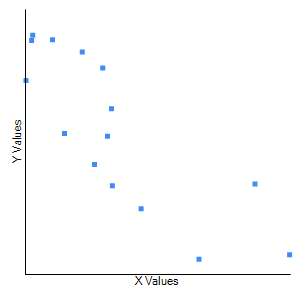 |
| --- | --- | --- |
|  |  |  |

| X - M_x_ | Y - M_y_ | (X - M_x_)^2^ | (Y - M_y_)^2^ | (X - M_x_)(Y - M_y_) |
| --- | --- | --- | --- | --- |
| -444.333  -450.333  -335.333  -172.333  -59.333  -11.333  -33.333  777.667  968.667  469.667  151.667  -6.333  -104.333  -269.333  -481.333  Mx: 481.333 | 107.133  101.133  102.133  88.133  70.133  24.133  -6.867  -60.867  -140.867  -145.867  -88.867  -62.867  -38.867  -3.867  56.133  My: 163.867 | 197432.111  202800.111  112448.444  29698.778  3520.444  128.444  1111.111  604765.444  938315.111  220586.778  23002.778  40.111  10885.444  72540.444  231681.778  Sum: 2648957.33 | 11477.551  10227.951  10431.218  7767.484  4918.684  582.418  47.151  3704.751  19843.418  21277.084  7897.284  3952.218  1510.618  14.951  3150.951  Sum: 106803.733 | -47602.911  -45543.711  -34248.711  -15188.311  -4161.244  -273.511  228.889  -47333.978  -136452.844  -68508.711  -13478.111  398.156  4055.089  1041.422  -27018.844  Sum: -434087.333 |

| **Result Details & Calculation** *X Values ∑ = 7220 Mean = 481.333 ∑(X - M_x_)^2^ = SS_x_ = 2648957.333  Y Values ∑ = 2458 Mean = 163.867 ∑(Y - M_y_)^2^ = SS_y_ = 106803.733  X and Y Combined N = 15 ∑(X - M_x_)(Y - M_y_) = -434087.333  R Calculation r = ∑((X - M_y_)(Y - M_x_))/√((SS_x_)(SS_y_))  r = -434087.333/√((2648957.333)(106803.733)) = -0.8161  Meta Numerics (cross-check) r = -0.8161* | Key  *X*: X Values *Y*: Y Values *M_x_*: Mean of X Values *M_y_*: Mean of Y Values *X - M*_x_ & *Y - M_y_*: Deviation scores *(X - M_x_*)^2^ & *(Y - M_y_)^2^*: Deviation Squared *(X - M_x_)(Y - M_y_*): Product of Deviation Scores |
| --- | --- |

The value of R is -0.8161.

This is a strong negative correlation, which means that high X variable scores are associated with low Y variable scores (and vice versa).

The value of R^2^, the coefficient of determination, is 0.666.

The P value is.000207. The result is significant at p <.05.

**Spearman's Rho Calculator**

The value of *r_s_* is -0.83929.

*r_s_* = -0.83929, *p* (2-tailed) = 9E-05.

By normal standards, the association between the two variables was considered statistically significant.

| X Values | Y Values | *X_Ra_* | *X_Ra_ - M_x_* | *Y_Ra_* | *Y_Ra_ - M_y_* | *Sum Diffs* |
| --- | --- | --- | --- | --- | --- | --- |
| 37  31  146  309  422  470  448  1259  1450  951  633  475  377  212  0 | 271  265  266  252  234  188  157  103  23  18  75  101  125  160  220 | 3.00  2.00  4.00  6.00  8.00  10.00  9.00  14.00  15.00  13.00  12.00  11.00  7.00  5.00  1.00 | -5.00  -6.00  -4.00  -2.00  0.00  2.00  1.00  6.00  7.00  5.00  4.00  3.00  -1.00  -3.00  -7.00 | 15.00  13.00  14.00  12.00  11.00  9.00  7.00  5.00  2.00  1.00  3.00  4.00  6.00  8.00  10.00 | 7.00  5.00  6.00  4.00  3.00  1.00  -1.00  -3.00  -6.00  -7.00  -5.00  -4.00  -2.00  0.00  2.00 | -35.00  -30.00  -24.00  -8.00  0.00  2.00  -1.00  -18.00  -42.00  -35.00  -20.00  -12.00  2.00  0.00  -14.00 |

| Calculation  R = CoVariance/(X_Ra_ St. Dev. * Y_Ra_ St. Dev.)  Key  X_Ra_ = Ranks of X Values; Y_Ra_ = Ranks of Y Values X_Ra_ - M_x_ = X rank minus mean of X ranks Y_Ra_ - M_y_ = Y rank minus mean of Y ranks Sum Diffs = (X_Ra_ - M_x_) * (Y_Ra_ - M_y_) | Result Details *X Ranks* Mean: 8 Standard Dev: 4.47  *Y Ranks* Mean: 8 Standard Dev: 4.47  *Combined* Covariance = -235/14 = -16.79 R = -16.79/(4.47 * 4.47) = -0.839 |
| --- | --- |

*r_s_* = -0.83929, *p* (2-tailed) = 9E-05.

By normal standards, the association between the two variables was considered statistically significant.

Table S6. The Factor for **Correlation Coefficient Determination.**

| year | DDS (+)^*^ | DDS (-)^*^ | sum AD (-)^*^ | year | DDS (+) | DDS (-) | sum AD (+)^*^ | TOTAL | sum DDS (+) | FACTOR^*^ |
| --- | --- | --- | --- | --- | --- | --- | --- | --- | --- | --- |
| 2005 | 290 | 417 | 707 | 2005 | 18 | 19 | 37 | 744 | 308 | **271** |
| 2006 | 302 | 363 | 665 | 2006 | 20 | 37 | 57 | 722 | 322 | **265** |
| 2007 | 317 | 332 | 649 | 2007 | 22 | 51 | 73 | 722 | 339 | **266** |
| 2008 | 310 | 312 | 622 | 2008 | 22 | 58 | 80 | 702 | 332 | **252** |
| 2009 | 300 | 283 | 583 | 2009 | 19 | 66 | 85 | 668 | 319 | **234** |
| 2010 | 270 | 286 | 556 | 2010 | 25 | 82 | 107 | 663 | 295 | **188** |
| 2011 | 255 | 268 | 523 | 2011 | 35 | 98 | 133 | 656 | 290 | **157** |
| 2012 | 238 | 241 | 479 | 2012 | 39 | 135 | 174 | 653 | 277 | **103** |
| 2013 | 195 | 248 | 443 | 2013 | 34 | 172 | 206 | 649 | 229 | **23** |
| 2014 | 172 | 236 | 408 | 2014 | 25 | 190 | 215 | 623 | 197 | **18** |
| 2015 | 167 | 168 | 335 | 2015 | 26 | 242 | 268 | 603 | 193 | **75** |
| 2016 | 154 | 149 | 303 | 2016 | 33 | 255 | 288 | 591 | 187 | **101** |
| 2017 | 143 | 115 | 258 | 2017 | 37 | 268 | 305 | 563 | 180 | **125** |
| 2018 | 132 | 87 | 219 | 2018 | 45 | 292 | 337 | 556 | 177 | **160** |
| 2019 | 114 | 40 | 154 | 2019 | 46 | 334 | 380 | 534 | 160 | **220** |
| 2020 | 109 | 4 | 113 | 2020 | 32 | 352 | 384 | 497 | 141 | **243** |
| Sum | 3468 | 3549 | 7017 | Sum | 478 | 2651 | 3129 | 10146 |  |  |
| Mean | 216.75 | 221.81 | 438.56 | Mean | 29.88 | 165.69 | 195.56 | 634.13 |  |  |
| SD^**^ | 76.04 | 117.71 | 190.18 | SD | 9.03 | 112.31 | 119.15 | 309.33 |  |  |
| 95% CI^**^ | 2.53 | 3.87 | 4.45 | 95% CI | 0.81 | 4.28 | 4.18 | 8.63 |  |  |
|  | 214.22 | 217.94 | 434.11 |  | 29.06 | 161.41 | 191.39 | 625.50 |  |  |
|  | 219.28 | 225.69 | 443.01 |  | 30.69 | 169.96 | 199.74 | 642.75 |  |  |

^*^Four groups were classified: DDS-prescribed (+) subjects, DDS-unprescribed (-) subjects, AD-diagnosed (+) subjects, and AD-undiagnosed subjects (-) subjects. FACTOR: The Factor = ㅣDDS (+) – sum AD (+) ㅣ

^**^, Standard Deviation (SD), Confident Interval (CI).

5.4. Asthma and lung function trajectories leading to COPD

### Fig. S13. Asthma and lung function trajectories leading to COPD

**
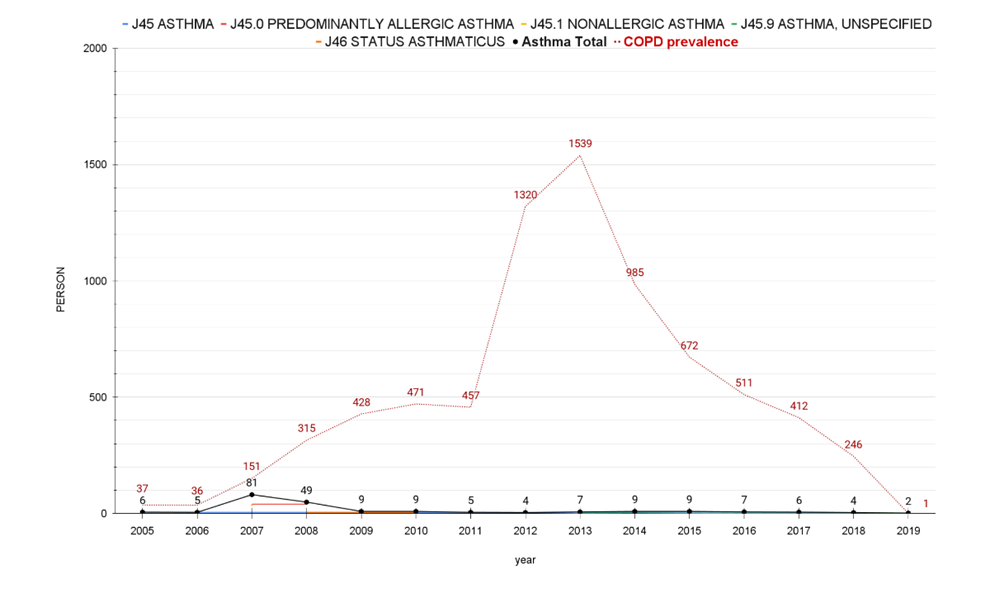
**

We reviewed the prevalence of asthma patients from the ICD-10 codes ASTHMA (J45), PREDOMINANTLY ALLERGIC ASTHMA (J45.0), NONALLERGIC ASTHMA (J45.1), ASTHMA, UNSPECIFIED (J45.9), and STATUS ASTHMATICUS (J46).

We reviewed the prevalence of chronic obstructive pulmonary disease (COPD) patients: OTHER CHRONIC OBSTRUCTIVE PULMONARY DISEASE (J44), CHRONIC OBSTRUCTIVE PULMONARY DISEASE, UNSPECIFIED (J44.9), and BRONCHIECTASIS (J47). COPD is regarded as one of the foremost causes of morbidity and mortality worldwide, yet its proper diagnosis remains a challenge ^41^.

The prevalence of asthma–COPD was not associated at all. In addition, bronchiectasis only developed in two to three cases of COPD. Therefore, we appreciate that they diagnosed COPD correctly.

Section 7. Safety

Dapsone's adverse reactions are well documented. Consequently, the medical staff managed all HD patients using this drug on Sorok Island to avoid its well-described side effects as much as possible. The adverse reactions associated with this drug include the clinical triad of fever, rash, and systemic involvement, which can cause severe organ dysfunction (most commonly in the liver and the hematologic system). Dapsone hypersensitivity can also lead to leukocytosis and eosinophilia, resembling a mononucleosis infection. The drug also has hematological effects, such as hemolytic anemia and methemoglobinemia. Other less frequent adverse reactions include hepatitis/liver toxicity, cholangitis, colitis, thyroiditis, pancreatitis and pleural effusion: acute renal failure: myocarditis, dapsone-induced hypersensitivity syndrome-associated complete atrioventricular block, myocardial injury: pneumonitis, pneumonia or multiple organ failure.

References

1. J. S. H. Kim, *Leprosy in Korea: A global history*. (University of California, Los Angeles, 2012).

2. S. H. K. Jane, Leprosy and Citizenship in Korea under American Occupation (1945~1948). *Sahak Yonku : The Review of Korean History*, 253-283 (2010).

3. M. Anthony. (LWW, 2019).

4. J.-h. Lee, H. K. An, M.-G. Sohn, P. Kivela, S. Oh, 4,4′-Diaminodiphenyl Sulfone (DDS) as an Inflammasome Competitor. *International Journal of Molecular Sciences* **21**, 5953 (2020).

5. J. W. Han *et al.*, Overview of the Korean Longitudinal Study on Cognitive Aging and Dementia. *Psychiatry Investig* **15**, 767-774 (2018).
